# CD4^+^ Resident Memory T Cells Mediate Long-Term Local Skin Immune Memory of Contact Hypersensitivity in BALB/c Mice

**DOI:** 10.3389/fimmu.2020.00775

**Published:** 2020-05-19

**Authors:** Akihiko Murata, Shin-Ichi Hayashi

**Affiliations:** Division of Immunology, Department of Molecular and Cellular Biology, School of Life Science, Faculty of Medicine, Tottori University, Yonago, Japan

**Keywords:** tissue-resident memory T cells, helper T cells, killer T cells, local skin memory, contact hypersensitivity, allergic contact dermatitis

## Abstract

In allergic contact dermatitis (ACD) and contact hypersensitivity (CHS), the healed skin shows greater swelling than the naïve skin in the same individual upon re-exposure to the same hapten. This “local skin memory” (LSM) in healed skin was maintained for a prolonged period of time and mediated by skin CD8^+^-resident memory T (T_RM_) cells in C57BL/6 mice. However, the number of CD4^+^ T cells is elevated in ACD-healed human skin, and the contribution of CD4^+^ T_RM_ cells to the formation of LSM currently remains unclear. We herein demonstrated that immediately after CHS subsided, the healed skin in BALB/c mice showed an accumulation of hapten-specific CD4^+^ and CD8^+^ T_RM_ cells, with a predominance of CD4^+^ T_RM_ cells. The presence of CD4^+^ or CD8^+^ T_RM_ cells in the healed skin was sufficient for the induction of a flare-up reaction upon a re-challenge. The CD4^+^ and CD8^+^ T_RM_ cells both produced interferon-γ and tumor necrosis factor early after the re-challenge. Moreover, while CD8^+^ T_RM_ cells gradually decreased over time and were eventually lost from the healed skin at 40–51 weeks after the resolution of CHS, the CD4^+^ T_RM_ cell numbers remained elevated during this period. The present results indicate that the long-term maintenance of LSM is mediated by CD4^+^ T_RM_ cells, and thus CD4^+^ T_RM_ cells are an important target for the treatment of recurrent human ACD.

## Introduction

Allergic contact dermatitis (ACD) and its animal model, contact hypersensitivity (CHS), are T cell-mediated delayed-type hypersensitivity reactions caused by sensitization and secondary epicutaneous exposure (elicitation or challenge) to contact allergens (e.g., haptens and metallic cations) (1–4). A flare-up (or retest) reaction of ACD often occurs on previously affected sites (4, 5). In guinea pigs, inflammation in a CHS-healed region was found to be more severe than that in the previously unaffected region in the same individual upon re-exposure to the same hapten (6). These findings suggested the existence of local immune memory in healed skin, named local skin memory (LSM), which may be mediated by hapten-specific locally persisting memory T cells. LSM was then shown to be a long-term immune memory that persisted for at least 1 year in BALB/c mice (7, 8). LSM is now considered to be mediated by tissue-resident memory T (T_RM_) cells based on the findings of studies using C57BL/6 mice (9–11).

CD4^+^ and CD8^+^ T_RM_ cells are a subset of αβ T cell receptor (TCR)^+^ memory T cells that fundamentally differ from their circulating counterparts and are characterized by their long-term residency within virtually all organs and unique transcriptional profiles (12–16). T_RM_ cells differentiate within tissues from activated T cells (or T_RM_ precursor cells), are maintained by local cytokine signals (17–22), and thus are concentrated in previously infected or inflamed tissues. The major function of T_RM_ cells is to provide protective immunity against pathogen re-entry into the same tissue; however, when generated in the context of allergy or autoimmune diseases, they are considered to cause disease relapse.

Skin T_RM_ cells have been characterized in various infections and diseases in both human and mouse models. Healthy and affected skin are populated by CD4^+^ and CD8^+^ T cells with various expression levels of CD69 and the integrin α_E_ chain (CD103) (17, 23–28). Sessile skin T_RM_ cells in previously inflamed skin are generally detected in CD69^+^CD103^+/−^ compartments in both CD8^+^ and CD4^+^ T cells, and CD69^−^ compartments represent recirculating populations (25, 29–32). CD8^+^ T_RM_ cells are more likely to preferentially localize in the epidermal layer, whereas CD4^+^ T_RM_ cells favor localization in the dermis, and the T_RM_ cell population that predominantly accumulates in inflammation-resolved skin appears to vary according to pathogens and diseases (11, 23, 25, 31, 33, 34).

Recent mechanistic analyses of LSM were conducted with C57BL/6 mice, and the findings obtained showed that CD8^+^ T_RM_ cells predominantly accumulated in CHS-healed skin (9–11). A previous study demonstrated that C57BL/6 healed skin contained elevated numbers of CD8^+^ T_RM_ cells, but not their CD4^+^ counterparts, which induced flare-up reactions in healed skin upon a re-challenge (hereafter referred to as the LSM response) (11). However, this is inconsistent with the finding showing that CD4^+^ T cells dominantly remained and persisted in ACD-healed human skin (35). Therefore, it currently remains unclear whether CD4^+^ T cells in healed skin contribute to the formation of LSM. Moreover, the number of CD8^+^ T_RM_ cells in the CHS-healed skin of C57BL/6 mice was found to progressively and markedly decrease for up to 12 months (11); however, the mechanisms by which LSM is maintained over a prolonged period of time currently remain unclear.

In the present study, we examined LSM in BALB/c mice with long-term LSM. The CHS-healed skin of BALB/c mice accumulated both CD4^+^ and CD8^+^ T_RM_ cells, with a predominance of CD4^+^ T_RM_ cells. We found that the presence of CD4^+^ or CD8^+^ T_RM_ cells in healed skin induced the LSM response upon a re-challenge, suggesting that they redundantly mediate LSM. Moreover, while CD8^+^ T_RM_ cells gradually decreased and disappeared from the skin by 56 weeks after the 1^st^ challenge, the increase in CD4^+^ T cell numbers was maintained during this period. Thus, CD4^+^ T_RM_ cells play a prominent role in the long-term maintenance of LSM.

## Materials and Methods

### Ethics Statement

All experiments were approved by and performed in strict accordance with the guidelines of the Animal Care and Use Committee of Tottori University (approval numbers 14-Y-6, 16-Y-3, and 19-Y-51).

### Mice

BALB/c mice (CLEA Japan, Tokyo, Japan) and C.Cg-Tg(DO11.10)10Dlo/J (BALB/c-DO11.10) mice (36) were bred and maintained in a specific pathogen-free facility in Tottori University. The BALB/c-DO11.10 mice were kindly provided by Dr. Toshinori Nakayama (Chiba University, Chiba, Japan) with permission from Dr. Kenneth M. Murphy (Washington University, St. Louis, MO, USA). C.B-17-*Prkdc*^*scid*/*scid*^ (C.B-17 SCID) and CAnN.Cg-*Foxn1*^*nu*/*nu*^ (BALB/c-*Foxn1*^*nu*/*nu*^) mice (Charles River Laboratories Japan, Kanagawa, Japan) were purchased and maintained in the facility.

### Haptens and Inhibitors

2,4,6,-Trinitrochlorobenzene (TNCB) (Tokyo Chemical Industry, Tokyo, Japan) and 4-ethoxymethylene-2-phenyl-2-oxazolin-5-one (oxazolone; Ox) (Sigma-Aldrich Japan, Tokyo, Japan) were dissolved in vehicle (acetone/olive oil = 4:1) (FUJIFILM Wako Pure Chemical Corporation, Osaka, Japan). Fluorescein isothiocyanate isomer I (FITC) (Sigma-Aldrich Japan) was dissolved in vehicle (acetone/dibutyl phthalate = 1:1). Haptens were prepared at each use.

The sphingosine-1-phosphate (S1P) receptor agonist, fingolimod (hydrochloride) (FTY720) (Cayman Chemical, Ann Arbor, MI, USA), was initially dissolved in ethanol. At the time of administration, 60 μg of FTY720 was dissolved in 300 μl phosphate-buffered saline (PBS) by intense pipetting with a 26G needle and syringe and was then injected i.p. into each mouse (approximately 2 mg/kg). A control injection with the same volume of ethanol (3.0 μl) in 300 μl PBS was performed.

The Jak1/2 inhibitor ruxolitinib (Rux) (ChemScene, Monmouth Junction, NJ, USA) and NF-κB inhibitor BAY (Tokyo Chemical Industry) were dissolved in dimethyl sulfoxide (DMSO) (Merck KGaA, Darmstadt, Germany). They were then diluted with distilled water containing 30% polyethylene glycol (PEG) 400 and 5% Tween 80 (FUJIFILM Wako) (hereafter PEG/Tween) for administration or with acetone for topical application. In **Figure 8F**, 240 μg Rux + 180 μg BAY (10.8 μl in total) + 150 μl PEG/Tween (approximately 8 mg/kg Rux and 6 mg/kg BAY) was injected i.p. into each mouse. Simultaneously, 10 μl of 0.5% Rux + 0.3% BAY in acetone (20% DMSO) was topically applied to both sides of both ears (20 μl/ear). In **Figure 8G**, 600 μg Rux + 600 μg BAY (32 μl in total) + 200 μl PEG/Tween (approximately 20 mg/kg each) was injected i.p. into each mouse. A control injection and a topical application were performed with the same volume of DMSO dissolved in each vehicle.

### CHS

On day−7 or−6, the right ears of the mice were sensitized with 20 μl of haptens (10 μl, both sides of the ear) under isoflurane anesthesia. In some experiments, sensitization with 100 μl of haptens was performed on shaved back skin. On day 0, the right ears were challenged with 20 μl of haptens and the left ears received 20 μl of vehicle (10 μl, both sides of the ear). After more than 35 days, the naïve and the healed ears were both re-challenged with 20 μl of haptens. In some experiments, both ears were re-re-challenged with 20 μl haptens at 35 days after the re-challenge. The experiments were conducted with 1% (40.4 mM) TNCB unless otherwise noted. Ear thickness was measured with the dial thickness gauge G-1A (Peacock) under isoflurane anesthesia. Hapten applications and measurements were conducted between 9:00 and 16:00 (for 2 days after the 1^st^ challenge, re-challenge, or re-re-challenge, ear thickness was measured every 22–26 h from the challenge time).

### Flow Cytometry on Isolated Ear Skin Cells

The ears were minced in 1 ml minimum essential medium α (Thermo Fisher Scientific-Gibco, Grand Island, NY, USA) supplemented with 2–3 mg/ml collagenase, 1–2 mg/ml hyaluronidase, 0.1 mg/ml DNAse I (FUJIFILM Wako), and antibiotics (penicillin and streptomycin, Meiji Seika, Tokyo, Japan) in microtubes. In some experiments, 20 μg/ml brefeldin A (Cayman Chemical) (to all samples) and 1.5 μg/ml ionomycin (Cayman Chemical) + 80 ng/ml phorbol 12-myristate 13-acetate (PMA) (AdipoGen Life Sciences, San Diego, CA, USA) (optional) were added to the digestion enzyme solution. The samples were incubated with constant rotation at 37°C for 3.5 h in an incubator with the mini rotator Bio RS-24 (Biosan, Riga, Latvia) unless otherwise stated. Subsequent operations were conducted on ice. Between each step, the cells were washed with excess stain buffer (Hank's solution; Nissui Pharmaceutical, Tokyo, Japan) containing 2.5% heat-inactivated fetal bovine serum (FBS) (Gibco) and 0.02% NaN_3_, and then centrifuged. The ear pieces were mashed with the plungers of 1-ml syringes on 70-μm cell strainers, and cells were collected with a stain buffer. The cells were then treated with Tris-NH_4_Cl for hemolysis (3 min) and collected through 35-μm cell strainers. The cells were incubated with 33% rabbit serum (Gibco) and 5 μg/ml purified anti-CD16/32 (2.4G2) for blocking. The cells were then stained with mAbs [combination of anti-CD3ε-FITC or -phycoerythrin (PE) (145-2C11, Thermo Fisher Scientific-eBioscience), CD4-PE or -PerCP-Cy5.5 (RM4-4, eBioscience or BioLegend, San Diego, CA), CD8α-PerCP-Cy5.5 (53-6.7, BioLegend), CD8β-FITC (H35-17.2, eBioscience), CD11b-FITC (M1/70, eBioscience), CD45-PerCP-Cy5.5 (30-F11, eBioscience), CD69-PE or -PerCP-Cy5.5 (H1.2F3, eBioscience or BioLegend), CD103-FITC (2E7, BioLegend), or Ly6G-biotin (1A8, BioLegend)]. Biotinylated mAbs were detected by further staining with streptavidin-PE (SouthernBiotech, Birmingham, AL, USA). The cells were finally stained with propidium iodide (Sigma-Aldrich Japan) and analyzed using the flow cytometer EPICS XL (Beckman Coulter, Brea, CA, USA) and WinMDI ver. 2.9 software. Each gating was made based on negative control staining with appropriate isotype-matched control mAbs [Tonbo Biosciences (San Diego, CA, USA), BioLegend, or eBioscience].

Regarding intracellular staining, after blocking and surface staining, the cells were fixed with 4% paraformaldehyde (PFA) (FUJIFILM Wako) and permeabilized with Perm Buffer (Tonbo Biosciences). The cells were then stained with anti-interferon (IFN)γ-PE (XMG1.2, eBioscience), tumor necrosis factor (TNF)-FITC (MP6-XT22, BioLegend), interleukin (IL)-4-Alexa488 (11B11, BioLegend), Foxp3 (3G3, Tonbo Biosciences), rat IgG1 control-PE (RTK2071, BioLegend) and -FITC (HRPN, Tonbo Biosciences), and/or mouse IgG1 control-PE (MOPC-21, Tonbo Biosciences) in Perm Buffer. The cells were then washed and analyzed as described above.

### Antibody-Mediated Cell Depletion and Adoptive Cell Transfer Assays

In antibody-mediated T cell depletion, the mice were injected i.p. with 97–100 μg of anti-CD4 [GK1.5, LEAF/Ultra-LEAF (BioLegend) or *InVivo*MAb (Bio X Cell, West Lebanon, NH, USA)] and/or anti-CD8α [53-6.7, LEAF/Ultra-LEAF (Biolegend)], or anti-Thy1.2 mAbs [30H12, *InVivo*MAb (Bio X Cell)] in 200 μl of sterile PBS as indicated in each figure.

Regarding the transfer of sensitized lymph node (LN) cells into C.B-17 SCID mice, the BALB/c mice were sensitized on the ears (20 μl/ear), shaved back, and the thorax–abdomen (100 μl each) with 1% TNCB on day−6. On day−1, LN cells (ear draining, axillary, brachial, and inguinal LNs) were collected by homogenizing with slide glasses in autoMACS running buffer (Miltenyi Biotec, Tokyo, Japan). After passing through nylon meshes, the cells were incubated with Tris-NH_4_Cl for hemolysis. After washing, some cells were separated for the transfer of whole LN cells. The remaining cells were divided into two groups, incubated with 33% rabbit serum for blocking, and then stained with purified anti-CD4 (GK1.5) or anti-CD8α (53-6.7) mAbs (Tonbo Biosciences). The cells were then incubated with rabbit anti-rat IgG-biotin (Vector Laboratories, Burlingame, CA, USA) and Streptavidin Particles Plus–DM (BD Biosciences, San Jose, CA, USA). The positive cells were removed by two rounds of incubation on the cell separation magnet IMag (BD Biosciences). Purified negative fractions (ΔCD4 and ΔCD8) and whole LN cell fractions were washed three times with excess sterile PBS, suspended in sterile PBS, and injected i.v. into the mice with 27G needles and 0.5-ml syringes (350 μl of the suspension per mouse).

Regarding the transfer of enriched splenic CD4^+^ and/or CD8^+^ T cells into DO11.10 mice, wild-type (WT) naïve BALB/c splenocytes were collected and hemolyzed with Tris-NH_4_Cl. After blocking with rabbit serum, the cells were stained with GK1.5 or 53-6.7 + common mAbs [anti-γδTCR (eBioGL3), B220 (RA3-6B2), CD11b (M1/70), CD11c (N418), F4/80 (BM8.1), CD16/32 (2.4G2), CD117 (ACK2), CD138 (281-2), and TER119 (eBioscience or Tonbo Biosciences)], rabbit anti-rat IgG-biotin + goat anti-hamster IgG-biotin (Vector Laboratories), and then Streptavidin Particles Plus–DM. The positive cells were removed with IMag, and the negatively enriched CD4^+^ and/or CD8^+^ T cells were injected i.v. as described above.

### Quantitative RT-PCR

The ears were preserved in RNAlater RNA stabilization solution (Qiagen, Hilden, Germany) at −80°C until use. According to the manufacturers' instructions, total RNA was extracted with the RNeasy Fibrous Tissue Mini Kit (Qiagen) and BioMasher II homogenizer (Nippi, Tokyo, Japan), and cDNA was synthesized with the PrimeScript RT reagent kit with a gDNA Eraser (Takara Bio, Shiga, Japan). qRT-PCR analyses [the shuttle PCR standard protocol in the Premix Ex Taq protocol (Takara Bio)] were performed using Light Cycler C480 (Roche, Basel, Switzerland) with dedicated 96-well plates. Each well contained cDNA (equivalent to 37.5 ng total RNA) diluted with Easy dilution (Takara Bio), 50% TB Green Premix Ex Taq II (Tli RNaseH Plus) (Takara Bio), 400 nM primers, and distilled water at 15 μl. Cp values were obtained with the Second Derivative Maximum Method using LightCycler software. Each gene expression level relative to *Hprt* expression in each cDNA sample was calculated with the ΔCt method. Pre-designed primers [Universal Probe Library Assay Design Center (Roche) or the Perfect Real Time Support System (Takara Bio)] were used, and their sequences were as follows (forward/reverse):

*Il1b* (5′-agttgacggaccccaaaag-3′/5′-agctggatgctctcatcagg-3′), *Il4* (5′-catcggcattttgaacgag-3′/5′-cgagctcactctctgtggtg-3′), *Il6* (5′-gctaccaaactggatataatcagga-3′/5′-ccaggtagctatggtactccagaa-3′), *Il7* (5′-ggaactgatagtaattgcccgaata-3′/5′-caccagtgtttgtgtgccttg-3′), *Il9* (5′-gcctctgttttgctcttcagtt-3′/5′-gcattttgacggtggatcat-3′), *Il13* (5′-cctctgacccttaaggagcttat-3′/5′-cgttgcacaggggagtct-3′), *Il15* (5′-gggatcctgctgtgtttggaa-3′/5′-cttaaggacctcaccagcaaggac-3′), *Il17a* (5′-cagggagagcttcatctgtgt-3′/5′-gctgagctttgagggatgat-3′), *Il17f* (5′-cccaggaagacatacttagaagaaa-3′/5′-caacagtagcaaagacttgaccat-3′), *Il18* (5′-caaaccttccaaatcacttcct-3′/5′-tccttgaagttgacgcaaga-3′), *Il22* (5′-tgacgaccagaacatccaga-3′/5′-aatcgccttgatctctccac-3′), *Il33* (5′-ggtgaacatgagtcccatca-3′/5′-cgtcacccctttgaagctc-3′), *Ifng* (5′-atctggaggaactggcaaaa-3′/5′-ttcaagacttcaaagagtctgaggta-3′), *Tgfb1* (5′-gtgtggagcaacatgtggaactcta-3′/5′-cgctgaatcgaaagccctgta-3′), *Tgfb2* (5′-ggagttcagacactcaacacaccaa-3′/5′-cagatcctgggacacacagca-3′), *Tgfb3* (5′-ccctggacaccaattactgcttc-3′/5′-ccttaggttcgtggacccatttc-3′), *Tnf* (5′-ctgtagcccacgtcgtagc-3′/5′-ttgagatccatgccgttg-3′), *Tslp* (5′-cagcttgtctcctgaaaatcg-3′/5′-aaatgttttgtcggggagtg-3′), *Cxcl1* (5′-gactccagccacactccaac-3′/5′-tgacagcgcagctcattg-3′), *Cxcl2* (5′-gaaaatcatccaaaagatactgaaca-3′/5′-ctttggttcttccgttgagg-3′), and *Hprt* (5′-tcctcctcagaccgctttt−3′/5′-cctggttcatcatcgctaatc -3′).

### Immunostaining of Ear Sections

The central region of the ears was cut and snap-frozen in optimal cutting temperature compound (Sakura Finetek Japan, Tokyo, Japan) with liquid nitrogen. Horizontal sections from the base of the ears (thickness of 7 μm) were cut with a cryostat and stored at −20°C until use. The sections were fixed in cold 4% PFA (3–5 min).

In immunohistochemistry (IHC), the fixed sections were incubated in 0.36% H_2_O_2_ in methanol (30 min) to block endogenous peroxidase, with 20% goat serum (FUJIFILM Wako) in block ace (DS Pharma Promo, Osaka, Japan) for blocking (100 min), then with primary mAbs (5 μg/ml, 120 min). Primary rat mAbs were purified anti-mouse CD3ε (17A2), CD4 (RM4-5), CD8α (53-6.7) + CD8β (H35-17.2), and Gr-1 (RB6-8C5) (Tonbo Biosciences). The sections were then incubated with ImmPRESS Goat anti-Rat IgG with polymer HRP (Vector Laboratories) (100 min). The mAbs were visualized with Impact Nova Red (Vector Laboratories) (10 min). The sections were counterstained with Hematoxylin QS (Vector Laboratories) and coverslipped with Malinol (Muto Pure Chemicals). The positive cells in the ear sections were counted along the cartilage (2.675 mm) under the microscope BX-60 (Olympus, Tokyo, Japan), and data were shown as cell numbers per millimeter. The positive cells in the epidermis, hair follicles, and sebaceous gland were counted as “in epidermis” and the cells in other parts as “in dermis.”

Regarding immunofluorescence (IF), the fixed sections were treated with the avidin/biotin blocking kit (Vector Laboratories) if biotinylated mAbs were used. The sections were incubated with 20% goat serum in block ace and then the primary mAbs (150 min) [combination of purified rabbit anti-CD3ε (SP7) (Novus Biologicals, Centennial, CO, USA), purified rat anti-CD4 or CD8α + CD8β (as described above), hamster anti-mouse γδTCR-biotin (eBioGL3), and biotinylated mouse anti-DO11.10 TCR (KJ1-26, Miltenyi Biotec)]. The mAbs were visualized with a combination of goat anti-rat IgG-Alexa555 (Cell Signaling Technology Japan, Tokyo, Japan), goat anti-rabbit IgG-DyLight488 (Vector Laboratories), and goat anti-hamster IgG-biotin and streptavidin-DyLight549 (Vector Laboratories). Regarding γδTCR IF, the sections were coverslipped with VECTASHIELD HardSet Antifade Mounting Medium with DAPI (Vector Laboratories), while for other IF, they were treated with the TrueVIEW Autofluorescence Quenching Kit (Vector Laboratories), stained with DAPI (Dojindo Laboratories, Kumamoto, Japan), and coverslipped with VECTASHIELD Vibrance Antifade Mounting Medium (Vector Laboratories).

All images were taken using the microscope BX-53 with appropriate mirror units and the digital camera DP73 and then analyzed with cellSens software (Olympus).

### Statistical Analysis

Each experiment was repeated more than twice with similar results and representative results were shown unless otherwise noted. Statistical analyses were performed using Microsoft Excel (for the paired or unpaired *t*-test) and IBM SPSS Statistics version 25 (for one-way ANOVA with *post-hoc* tests). The significance of differences was established at *p* < 0.05.

## Results

### Characterization of TNCB-Induced LSM in BALB/c Mice

**Figure 1:**
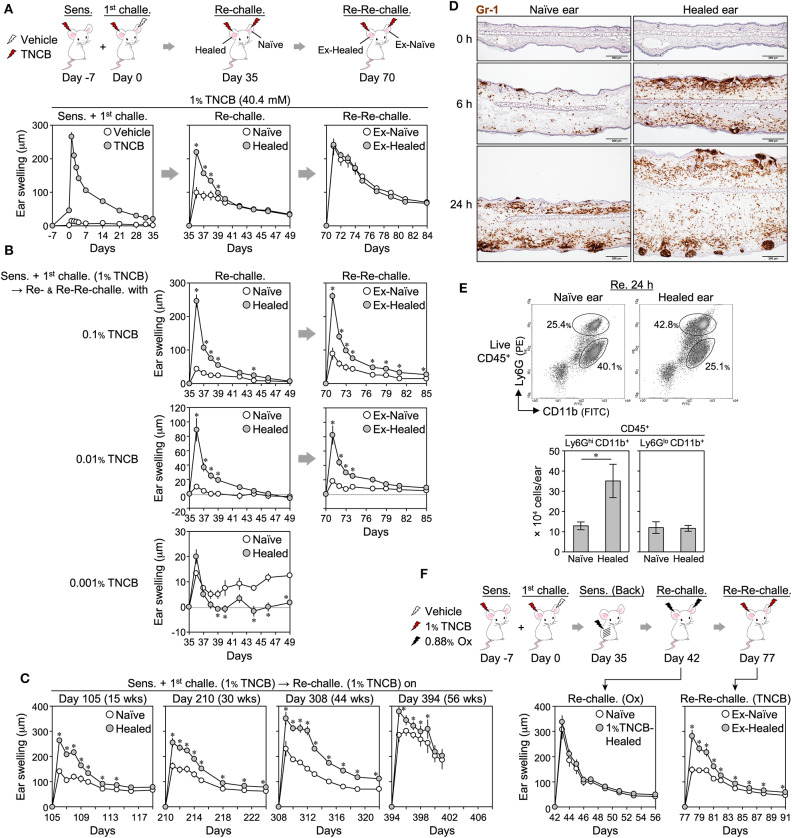
Characterization of 2,4,6,-trinitrochlorobenzene (TNCB)-induced local skin memory in BALB/c mice. **(A)** Protocol for contact hypersensitivity and ear swelling upon ear skin sensitization + the 1^st^ challenge (*n* = 9), re-challenge (*n* = 9), and re-re-challenge (*n* = 6). **(B)** Ear swelling upon the re-challenge and re-re-challenge with 0.1% (*n* = 5), 0.01% (*n* = 5), and 0.01% (*n* = 6) TNCB. **(C)** Ear swelling upon the re-challenge at 105–394 days after the 1^st^ challenge [day 105 (*n* = 6), day 210 (*n* = 7), day 308 (*n* = 7), and day 394 (*n* = 4)]. The data obtained on day 394 were from a one-time experiment. **(D)** Representative ear skin sections at 0, 6, and 24 h after the re-challenge (1% TNCB) stained with anti-Gr-1 mAb (Brown) (*n* = 3 each). Bars, 200 μm. **(E)** Representative histograms of isolated ear skin cells at 24 h after the re-challenge (1% TNCB) and the calculated numbers of neutrophils (Ly6G^hi^CD11b^+^) and other myeloid cells (Ly6G^lo^CD11b^+^) in each ear (*n* = 5). **(F)** Ear swelling upon back skin sensitization + a re-challenge with an irrelevant hapten (Ox) and upon a re-re-challenge with 1% TNCB (*n* = 6). All experiments were performed with BALB/c mice. Data are means ± SE. *P < 0.05 (two-tailed paired *t*-test).

We examined LSM in more detail using BALB/c mice showing the LSM response (7, 8). To induce CHS, the right ears of BALB/c mice were sensitized (day−7) and challenged (day 0) with the hapten 1% TNCB. Ear swelling peaked on day 1 and healed after 5 weeks (Figure 1A). On day 35, naïve (left) and healed (right) ears were both re-challenged with 1% TNCB. The healed ears showed significantly more swelling than the naïve ears for days after the re-challenge (Figure 1A), suggesting the formation of LSM in CHS-experienced skin. We referred to the greater swelling in re-challenged healed ears than in naïve ears as the LSM response. When we re-re-challenged ex-naïve and ex-healed ears with 1% TNCB at 5 weeks after the re-challenge (on day 70), the extent of swelling was similar in both ears, suggesting that LSM was formed in the naïve ears by the re-challenge (Figure 1A).

The LSM response occurred when the mice were sensitized on shaved back skin instead of ear skin (Supplemental Figure 1A). Furthermore, ear sensitization alone (day 0) induced weak swelling but not an effective LSM response in healed ears upon the challenge to both ears on day 35 (Supplemental Figure 1B). These results indicate that LSM only forms in the challenged site regardless of the route of sensitization. LSM responses similar to those with 1% TNCB were observed when other haptens (0.5% FITC and 0.88% Ox) were used (Supplemental Figure 1C).

When we re-challenged both ears with 0.1 and 0.01% of TNCB, but not with 0.001%, the CHS (with 1% TNCB)-healed ears still showed a marked LSM response (Figure 1B). Moreover, the peak swelling of the healed skin upon the re-challenge with 0.1 and 0.01% TNCB (Figure 1B) was markedly stronger than that in the 1^st^ CHS with the corresponding concentration of TNCB (Supplemental Figure 1D). Thus, TNCB-induced LSM enhanced the local antigen sensitivity and reactivity of the healed skin exposed to the low dose of TNCB (<0.1%), as shown previously with other haptens (8), but not the high dose of TNCB (1%).

Moreover, we found that LSM responses were still observed by the re-re-challenge with the same dose of TNCB (Figure 1B), in contrast to the re-re-challenged ears with 1% TNCB (Figure 1A), suggesting that the formation of LSM in re-challenged naïve skin requires a higher dose of the antigen challenge of more than 0.1% TNCB.

To confirm the long-term persistence of LSM (8), we re-challenged ears at 15, 30, 44, or 56 weeks after the 1^st^ challenge. In contrast to previous findings showing that the swelling response of the healed ears upon a re-challenge gradually diminished over time (8), the swelling of both naïve and healed ears upon a re-challenge increased over time in our experiments (Figure 1C). Nevertheless, the LSM response was observed even after 56 weeks (Figure 1C), confirming the long-term persistence of TNCB-induced LSM.

We found that the dermis of 1% TNCB re-challenged healed ears markedly accumulated Gr-1^+^ cells (neutrophils and monocyte-derived cells) within 6 h (Figure 1D). At 24 h, Gr-1^+^ cells accumulated in the hair follicles of the healed ears. Flow cytometry on isolated ear cells at 24 h after the re-challenge showed that the healed ears contained more neutrophils (Ly6G^Hi^CD11b^+^), but not other myeloid cells (Ly6G^Lo^CD11b^+^) (Figure 1E). Thus, the LSM response was characterized by the extensive accumulation of neutrophils.

To confirm the antigen specificity of TNCB-induced LSM, as shown previously with other haptens (6–8), we re-challenged both naïve and healed ears (harboring 1% TNCB-induced LSM) with Ox (Figure 1F) or FITC with or without secondary sensitization before the re-challenge (Supplemental Figure 1E). In all cases, the extent of swelling was similar in both ears, indicating that TNCB-induced LSM did not respond to unrelated haptens, and thus this was an antigen-specific response. Moreover, we found that upon the re-re-challenge with TNCB, Ox- or FITC-induced CHS-experienced skin showed a normal LSM response against TNCB (Figure 1F and Supplemental Figure 1E), indicating that original LSM was maintained after CHS induced by irrelevant antigens.

### CD4^+^ T_RM_ Cells Outnumber Their CD8^+^ Counterpart in BALB/c Healed Ears

**Figure 2:**
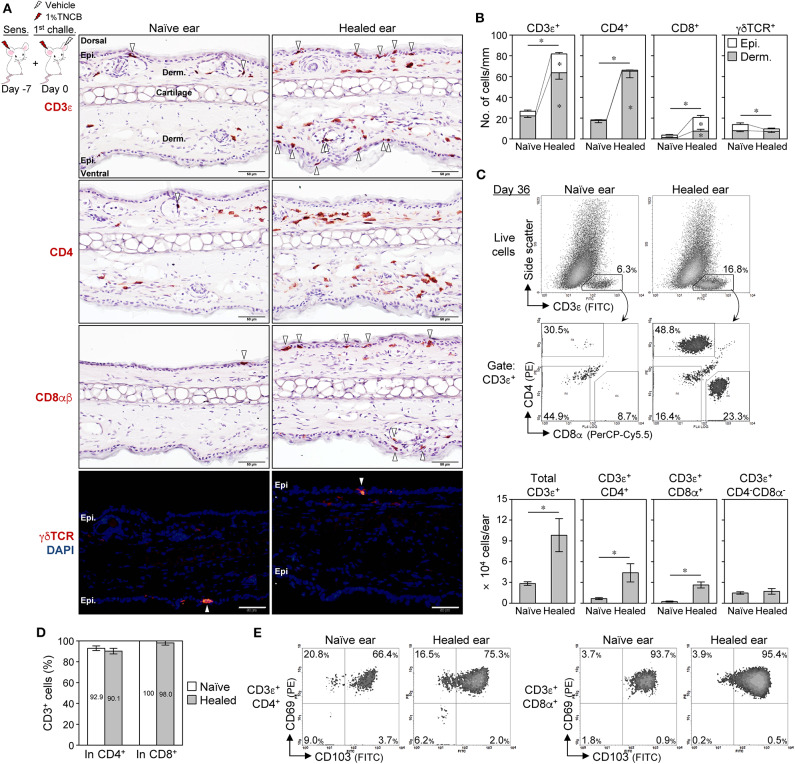
CD4^+^ T_RM_ cells outnumber CD8^+^ T_RM_ cells in BALB/c mouse contact hypersensitivity-healed skin. **(A,B)** Ear skin sections on day 35 after the 1^st^ challenge with 1% 2,4,6,-trinitrochlorobenzene (TNCB) were stained with the indicated mAbs. **(A)** Representative photomicrographs. Positive cells in the epidermis (including hair follicles and sebaceous glands) were marked by arrowheads. Bars, 50 μm. **(B)** The numbers of positive cells along the cartilage are shown as cells/mm (*n* = 6 each). The asterisks above the bars indicate significant differences in the total number of positive cells (epidermis + dermis) between naïve and healed ears. **(C)** Representative histograms of isolated ear skin cells on day 35 after the 1^st^ challenge (1% TNCB) and the calculated numbers of each fraction (*n* = 5) in each ear are shown. **(D)** Percent CD3^+^ cells in total CD4^+^ or CD8^+^ cells as assessed by an immunofluorescence analysis of ear sections on day 35 (*n* = 3). **(E)** CD69 and CD103 expression in CD4^+^ and CD8^+^ T cells in pooled ear skin cells (from five to six mice) on day 36 after the 1^st^ challenge with 1% TNCB. All experiments were performed with BALB/c mice. Bars represent means ± SE. *P < 0.05 (two-tailed paired *t*-test). Epi., epidermis; Derm., dermis.

A previous study reported that CD8^+^ T_RM_ cells predominantly accumulated in CHS-healed C57BL/6 mouse skin and were responsible for the LSM response (11). IHC on BALB/c mouse ear skin sections showed that the healed ears (day 35) contained higher numbers of CD3^+^ T cells both in the epidermis (including hair follicles and sebaceous glands) and the dermis than the naïve ears (Figures 2A,B). CD4^+^ and CD8^+^ cell numbers were both higher in the healed ears than in the naïve ears, with the CD4^+^ cells outnumbering the CD8^+^ cells (Figures 2A,B). The majority of CD4^+^ cells resided in the dermis of both ears, while CD8^+^ cells in the healed ears were in the epidermis and the dermis, with approximately 70% being in the epidermis (Figures 2A,B). Only a few dendritic epidermal γδ T cells were detected in BALB/c mice, which is consistent with previous findings (37, 38), and γδ T cells did not increase in the healed ears (Figures 2A,B). Flow cytometry on isolated ear skin cells also showed that the numbers of CD3^+^CD4^+^ and CD3^+^CD8^+^ T cells, but not CD3^+^CD4^−^CD8^−^ (including γδ T cells), increased in the healed ears on day 36 after the 1^st^ challenge (Figure 2C). A similar increase in CD4^+^ and CD8^+^ cell numbers in the healed ears was also observed in CHS with Ox or FITC (Supplemental Figures 2A,B) and CHS with 1% TNCB with back skin sensitization (Supplemental Figure 2C). The IHC showed that dermal CD4^+^ cells sometimes outnumbered dermal CD3^+^ cells. The IF analysis revealed that the CD8^+^ cells in the ear sections were almost exclusively CD3^+^, while CD4^+^ cells contained 10–20% of CD3^−^ non-T cells in both ears (Figure 2D and Supplemental Figure 2D).

Skin T_RM_ cells generally express CD69 and CD103, and sessile T_RM_ cells have been detected in CD69^+^ compartments in previously inflamed skin (25, 31). Moreover, skin T_RM_ cells, but not circulating T cells, were shown to be resistant to *in vivo* antibody-mediated cell depletion (20, 29). On day 36, approximately 70% of CD4^+^ T cells were CD69^+^CD103^+^ in both ears, while approximately 20% were CD69^+^CD103^−^, and the majority of CD8^+^ T cells were CD69^+^CD103^+^ regardless of epidermal and dermal localization (Figure 2E and Supplemental Figure 2E). Moreover, CD4^+^CD69^+^ and CD8^+^CD69^+^ T cells in the healed ear skin were resistant to *in vivo* antibody-mediated cell depletion with anti-CD4 or -Thy1.2 mAbs despite the effective depletion of splenic T cells (Supplemental Figure 3A). Furthermore, the CD4^+^ T cells in the healed skin contained ~15% of Foxp3^+^ regulatory T (Treg) cells (Supplemental Figures 3B,C), indicating that the majority of the skin CD4^+^ T cells were conventional T cells. These results suggest that the increased numbers of CD4^+^ and CD8^+^ T cells in the healed ears of BALB/c mice were T_RM_ cells.

**Figure 3:**
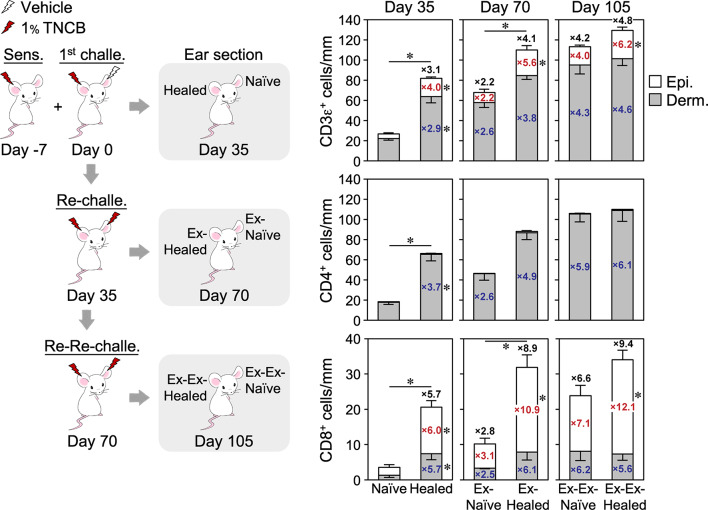
Increasing numbers of CD4^+^ and CD8^+^ T_RM_ cells form with recurrent contact hypersensitivity. The ear skin sections of a group of BALB/c mice at 35 days after the 1^st^ challenge (*n* = 6), re-challenge (*n* = 3), or re-re-challenge (*n* = 6) with 1% TNCB were analyzed by single immunohistochemistry staining with anti-CD3ε, CD4, or CD8α + CD8β mAbs. The numbers of positive cells along the cartilage are shown. Data for day 35 are the same as in Figure 2B. Bars represent means ± SE. *P < 0.05 between both ears on each day of the analysis (two-tailed paired *t*-test). The numbers inside and outside the bars indicate the fold change in each value [epidermis (red), dermis (blue), and total number (black)] relative to those in naïve ears on day 35.

The skin and other organs are large and flexible niches for CD8^+^ T_RM_ cells generated by pathogen infections, that is, repeated infections expand the T_RM_ cell population by inducing their proliferation as well as the formation of new T_RM_ cells from circulating T cells without displacing pre-existing populations (30, 39, 40). We investigated whether this was the case in CHS and CD4^+^ T_RM_ cells (Figure 3). After the resolution of 2^nd^ CHS by the re-challenge (day 70), the ex-naïve ears contained similar numbers of CD4^+^ and CD8^+^ T_RM_ cells as the healed ears on day 35. The ex-healed ears contained expanded epidermal, but not dermal CD8^+^ T_RM_ cells, as well as a higher number of dermal CD4^+^ T_RM_ cells than the 1^st^ CHS-healed ears. Since the ex-naïve and the ex-healed ears both showed a similar extent of swelling upon the re-re-challenge (Figure 1A), this result suggested that once T_RM_ cells are formed, the skin shows a constant swelling response upon re-exposure to the same concentration of the antigen regardless of how many T_RM_ cells are retained. After the resolution of the 3^rd^ CHS by the re-re-challenge (day 105), the ex-ex-healed ears contained higher numbers of dermal CD4^+^ and unchanged CD8^+^ T_RM_ cells than the ex-healed ears on day 70. The increased rates of CD4^+^ and CD8^+^ T_RM_ cells were the highest in the healed ears at the 1^st^ CHS (day 35) and gradually decreased in the 2^nd^ and subsequent CHS. These results demonstrated that both skin T_RM_ cell populations were expanded by repeated CHS, with CD8^+^ T_RM_ cells expanding exclusively in the epidermis and CD4^+^ T_RM_ cells in the dermis. Thus, the skin is also a flexible niche for CD4^+^ T_RM_ cells.

### Absence of LSM in T Cell-Deficient or DO11.10 Mice and Development of the LSM Response Without Recruitment of Effector T Cells

**Figure 4:**
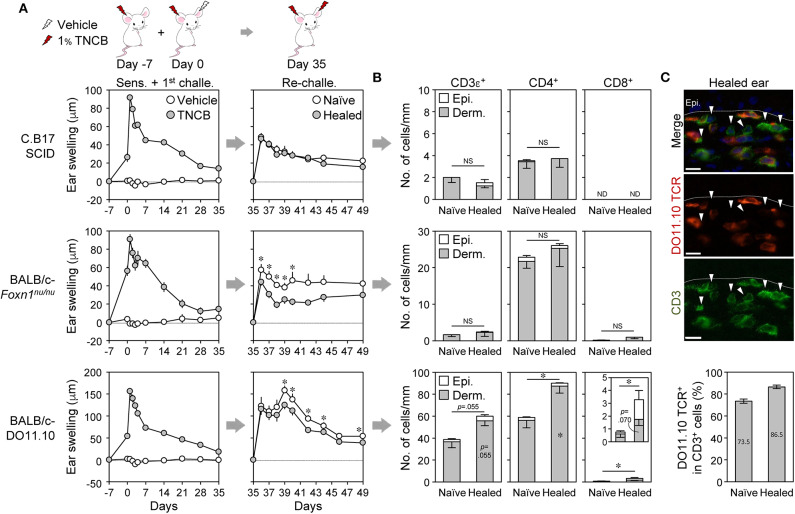
Absence of the local skin memory response in nude, SCID, and DO11.10 mice. **(A–C)** Contact hypersensitivity with 1% TNCB (as in Figure 1A) using SCID, nude (*Foxn1*^*nu*/*nu*^), and DO11.10 mice. **(A)** Ear swelling upon ear sensitization + the 1^st^ challenge (*n* = 10–11 mice) and re-challenge (*n* = 6–7 mice). **(B)** The numbers of CD3ε^+^, CD4^+^, and CD8αβ^+^ cells along the cartilage in ear skin sections on day 35 as analyzed by immunohistochemistry. SCID (*n* = 4), nude (*n* = 5), and DO11.10 (*n* = 5). **(C)** DO11.10 healed ears (day 35) were stained with anti-CD3ε (green) and anti-DO11.10 TCR (red). Arrowheads in the representative photomicrographs (original magnification, ×400) indicate CD3ε^+^DO11.10 TCR^−^ cells. Bars, 10 μm. The graph represents percent DO11.10 TCR^+^ cells in total CD3^+^ cells in naïve and healed ears (*n* = 3). Data represent means ± SE. *P < 0.05 (two-tailed paired *t*-test). NS, not significant. ND, not detected.

To confirm the involvement of T_RM_ cells in the LSM response of BALB/c mice, we initially analyzed C.B-17 SCID mice (lack T and B cells), BALB/c-nude mice (lack thymic T cells), and BALB/c-DO11.10 mice [in which many T cells express transgenic αβ TCR genes specific for the ovalbumin peptide on major histocompatibility complex (MHC) class II (I-A^d^)]. The SCID and the nude mice exhibited slight swelling with the 1^st^ CHS, and their healed ears did not show the LSM response upon a re-challenge with 1% TNCB (Figure 4A). The healed ears of SCID and nude mice did not accumulate T cells (Figure 4B and Supplemental Figure 4A) despite the nude mice having extrathymically differentiated T cells (41). The DO11.10 mice showed greater swelling than SCID and nude mice with the 1^st^ CHS, whereas the healed ears did not show the LSM response upon the re-challenge (Figure 4A). The healed ears accumulated a large number of CD4^+^ T cells and a small number of CD8^+^ T cells (Figure 4B and Supplemental Figure 4A), and the majority of the CD3^+^ cells in the healed ears were DO11.10 TCR^+^ cells (Figure 4C). These results suggest that the TNCB challenge in DO11.10 mice induced the recruitment of both DO11.10 TCR^+^ T cells and T cells with endogenous TCR, both of which were unable to mediate LSM. Collectively, the lack of an LSM response in these mice indicates that T cells are required for the initiation of the LSM response upon a re-challenge.

A previous study reported that skin T_RM_ cells alone induced the LSM response without the recruitment of effector T cells from the circulation in C57BL/6 mice (9). To examine whether this was the case in BALB/c mice, CHS-healed mice were treated with the S1P receptor agonist FTY720, which prevents lymphocyte egress from the LNs, before and after the re-challenge in order to block the recruitment of newly activated effector T cells to the inflamed skin. The treatment did not affect the ear swelling response upon the re-challenge but completely inhibited the recruitment of effector T cells in both ears at 24 h after the re-challenge (Supplemental Figures 4B,C). Moreover, the marked accumulation of neutrophils in the re-challenged ears was not inhibited by FTY720 (Supplemental Figure 4D). Therefore, the T_RM_ cells already present in the healed ears induced the LSM response in BALB/c mice.

### CD4^+^ and CD8^+^ T_RM_ Cells Redundantly Mediate the LSM Response

**Figure 5:**
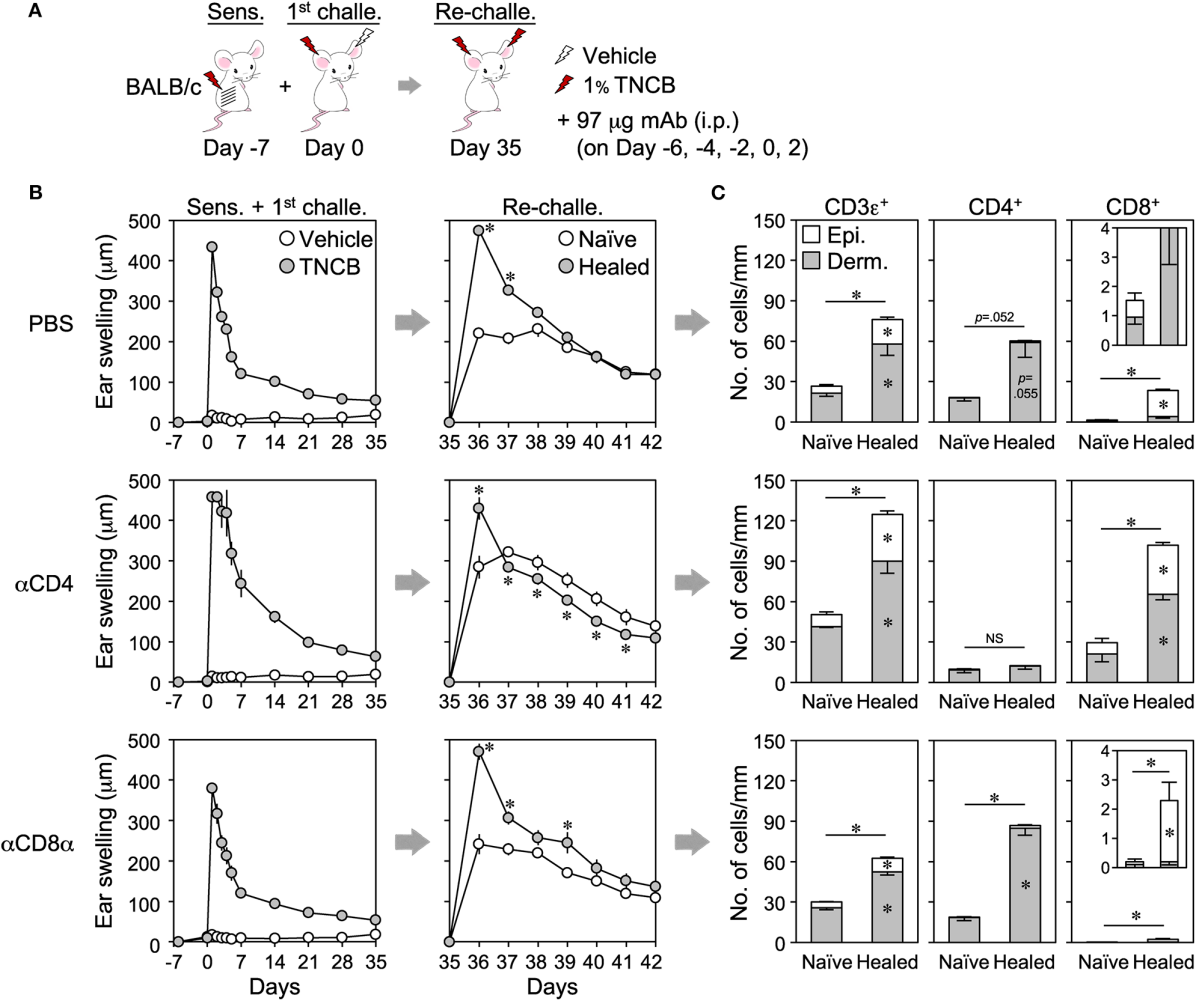
The antibody-mediated inhibition of CD4^+^ or CD8^+^ T_RM_ cell formation in healed ears does not suppress the local skin memory response. **(A)** BALB/c mice were sensitized on back skin and anti-CD4 (GK1.5) or CD8α (53–6.7) mAbs were injected on the indicated days in order to inhibit the accumulation of effector CD4^+^ or CD8^+^ T cells in inflamed ear skin during the 1^st^ contact hypersensitivity. Phosphate-buffered saline was injected as a control. **(B)** Ear swelling upon sensitization + the 1^st^ challenge and re-challenge (*n* = 6–7 each group). **(C)** The numbers of CD3ε^+^, CD4^+^, and CD8αβ^+^ cells along the cartilage in ear skin sections on day 35 as assessed by immunohistochemistry (*n* = 4 each group). Data represent means ± SE. *P < 0.05 (two-tailed paired *t*-test).

To identify which T_RM_ cells mediate the LSM response, back skin-sensitized BALB/c mice were injected with anti-CD4 (GK1.5) or -CD8α (53–6.7) mAb before and after the 1^st^ challenge in order to inhibit the accumulation of each T cell subset during the 1^st^ CHS and the formation of T_RM_ cells (by cell depletion and/or inhibition of T cell activation upon antigen presentation) (Figure 5A). Anti-CD4, but not anti-CD8α, mAb effectively depleted the circulating T cells, while both mAbs blocked the accumulation of T cells in the inflamed ear skin on day 1 after the 1^st^ challenge (Supplemental Figures 5A–D). CD4^+^ cell depletion resulted in more prolonged swelling in the 1^st^ CHS than in the PBS-treated control mice (Figure 5B). Upon the re-challenge, the extent of swelling in the healed ears was similar to that in the control mice, whereas that in the naïve ears was significantly greater (Figure 5B). Nevertheless, the peak swelling response of the healed ears at 1 day after the re-challenge was significantly greater than that of the naïve ears, suggesting the presence of the LSM response (Figure 5B). CD4^+^ cell depletion resulted in the accumulation of a larger number of CD8^+^ T_RM_ cells not only in the healed ears but also in the naïve ears but prevented the accumulation of CD4^+^ T_RM_ cells (Figure 5C and Supplemental Figure 5E). In contrast, injecting anti-CD8 mAb had no effect on the swelling responses throughout the experiment (Figure 5B), and the healed ears mainly accumulated CD4^+^ T_RM_ cells in addition to very few CD8^+^ T_RM_ cells (Figure 5C and Supplemental Figure 5E).

**Figure 6:**
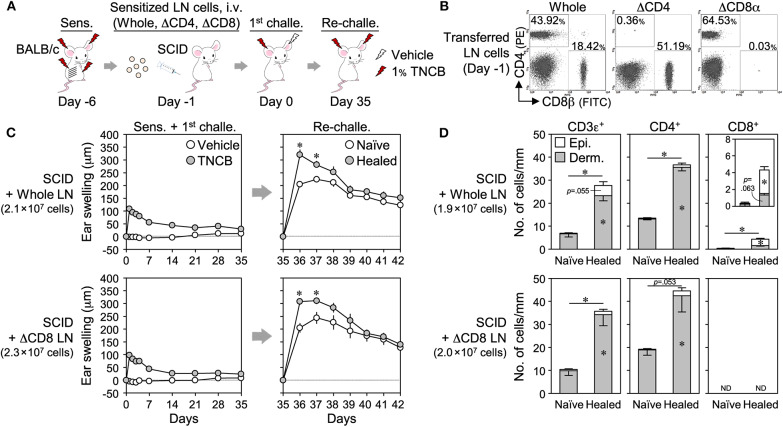
Adoptive transfer of CD8^+^ cell-depleted wild-type lymph node (LN) cells to SCID mice restores the local skin memory response. **(A)** Sensitized BALB/c LN cells (whole, ΔCD4, or ΔCD8) were adoptively transferred (i.v.) to SCID mice, and the 1^st^ challenge and the re-challenge with 1% TNCB were performed. **(B)** Removal efficiency of CD4^+^ or CD8^+^ cells in transferred LN cells on day−1 as assessed by flow cytometry. **(C)** Ear swelling upon 1^st^ challenge and re-challenge (*n* = 5 each group). **(D)** The numbers of CD3ε^+^, CD4^+^, and CD8αβ^+^ cells along the cartilage in ear skin sections on day 35 as assessed by immunohistochemistry. Whole (*n* = 3) and ΔCD8 (*n* = 4). The numbers of transferred LN cells per mouse were indicated in each figure. Data represent means ± SE. *P < 0.05 (two-tailed paired *t*-test).

We then adoptively transferred TNCB-sensitized BALB/c LN cells [whole, CD4^+^ cell-depleted (ΔCD4) or ΔCD8] into SCID mice, and the swelling responses upon 1^st^ challenge and re-challenge were assessed (Figures 6A,B). The transfer of whole LN cells restored the LSM response upon the re-challenge (Figure 6C) and the formation of CD4^+^ and CD8^+^ T_RM_ cells in healed ears (Figure 6D and Supplemental Figure 6A). The transfer of ΔCD8 LN cells also restored the LSM response, and the extent of swelling was similar to that in mice into which whole LN cells were transferred (Figure 6C). The healed ears contained increased numbers of CD4^+^ T_RM_ cells and no CD8^+^ T_RM_ cells (Figure 6D and Supplemental Figure 6A). We were unable to accurately assess ΔCD4 LN cell-transferred SCID mice because a small area with abnormally accumulated CD4^+^ and CD8^+^ T cells was observed in the healed ear sections of three out of five mice for an unknown reason(s) (Supplemental Figures 6B–D).

**Figure 7:**
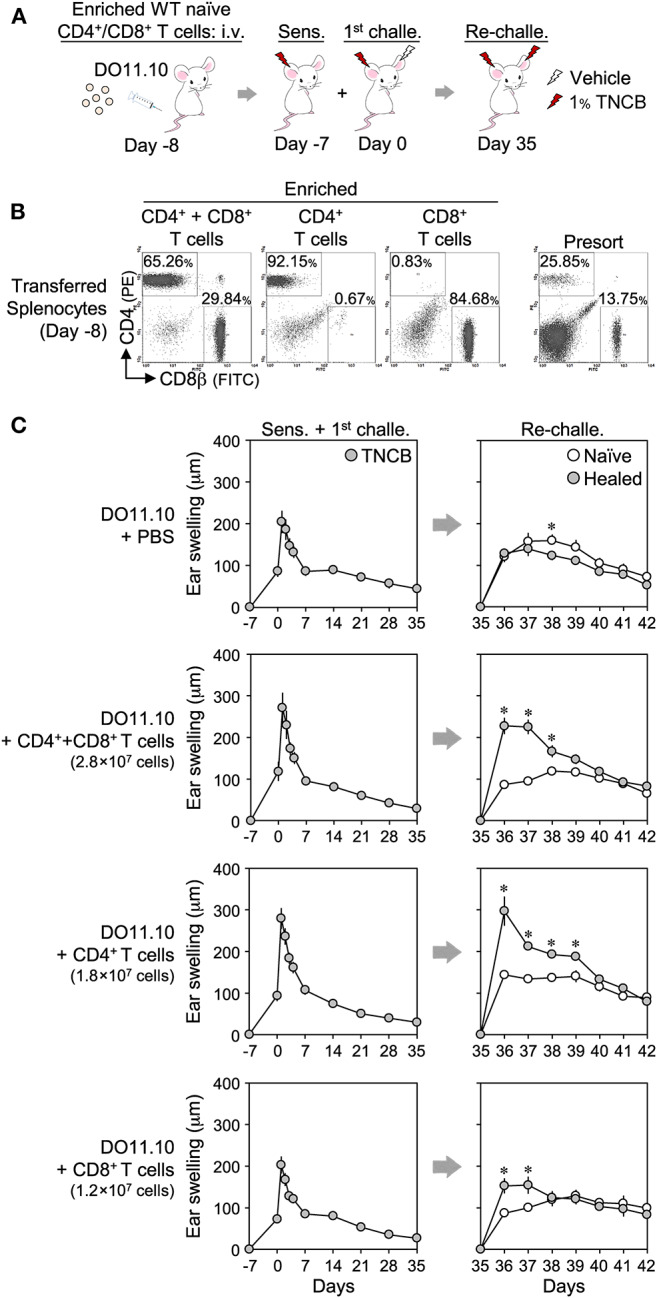
Adoptive transfer of enriched CD4^+^ and/or CD8^+^ splenic T cells to DO11.10 mice restores the local skin memory response. **(A)** Protocol. **(B)** CD4^+^ or CD8^+^ T cells were enriched from naïve BALB/c splenocytes using magnetic cell depletion. The enrichment efficiency of the transferred cells on day−8 was assessed by flow cytometry. **(C)** Ear swelling upon ear sensitization + the 1^st^ challenge and re-challenge. The numbers of transferred cells per mouse were indicated in each figure. CD4^+^ + CD8^+^ T cell-injected [1^st^ challenge (*n* = 5), re-challenge (*n* = 4)], other groups (*n* = 6 each). Data represent means ± SE. *P < 0.05 (two-tailed paired *t*-test).

We also adoptively transferred enriched splenic T cells from naïve BALB/c mice to DO11.10 mice lacking LSM (Figure 7). While the transfer of CD4^+^ or CD8^+^ T cells alone, or both, had no or a weak effect on the swelling response in the 1^st^ CHS, they restored the LSM response of the healed ears upon a re-challenge in DO11.10 mice.

Collectively, these results indicate that the presence of CD4^+^ or CD8^+^ T_RM_ cells in the healed ears was sufficient for the development of the LSM response, and thus these cells redundantly initiate the LSM response.

### CD4^+^ and CD8^+^ T_RM_ Cells Rapidly Produce IFNγ and TNF Upon a Re-challenge

**Figure 8:**
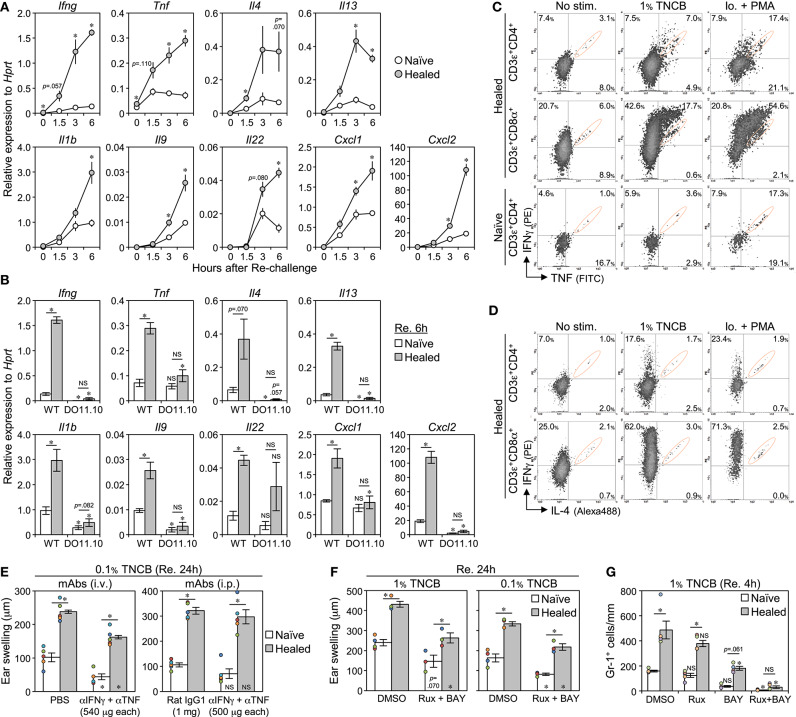
The early production of IFNγ and TNF by CD4^+^ and CD8^+^ T_RM_ cells is associated with the induction of the local skin memory response. **(A)** The mRNA levels of the indicated genes in whole naïve and healed ear extracts of BALB/c mice at 0, 1.5, 3, and 6 h after the re-challenge with 1% TNCB (on day 35 after the 1^st^ challenge) as assessed by qRT-PCR (*n* = 4 each time). **(B)** mRNA levels in the whole ear extract of DO11.10 mice at 6 h after the re-challenge with 1% TNCB (on day 37 after the 1^st^ challenge) (*n* = 4) were compared with those of BALB/c mice [data at 6 h in **(A)**]. **(C,D)** IFNγ, TNF, and IL-4 production in CD4^+^ and CD8^+^ T_RM_ cells (on day 39 after the 1^st^ challenge) as assessed by flow cytometry. No stimulation: naïve and healed ears underwent enzymatic digestion (3.5 h) with brefeldin A. 1% TNCB stimulation: ears (re-challenged 3.5 h before) underwent digestion with brefeldin A (3.5 h). Ionomycin + PMA stimulation: ears underwent enzymatic digestion (4.0 h) with brefeldin A, ionomycin, and PMA. Signals in orange circles were mostly a non-specific background, as shown in Supplemental Figure 7B. **(E)** 1^st^ contact hypersensitivity-healed BALB/c mice were injected (i.p. or i.v.) with anti-IFNγ + anti-TNF neutralizing mAbs, or control Rat IgG1 mAb or PBS, simultaneously with the re-challenge with 0.1% TNCB on day 35. Ear swelling at 24 h after the re-challenge is shown. Each experiment was performed once. **(F)** Ear swelling in BALB/c mice at 24 h after the re-challenge with 1 or 0.1% TNCB (on day 36 after the 1^st^ challenge). The mice were injected and topically treated with dimethyl sulfoxide (control) or Rux + BAY 2 h before the re-challenge. Injection: Rux (8 mg/kg) + BAY (6 mg/kg). Topical application: 0.5% Rux + 0.3% BAY. **(G)** The numbers of Gr-1^+^ cells along the cartilage in BALB/c ear skin sections at 4 h after the re-challenge (on day 36 after the 1^st^ challenge). The mice were injected with Rux and/or BAY (20 mg/kg each) at 1 h before the re-challenge (*n* = 4–5 each group). All graphs represent means ± SE. *P < 0.05 (two-tailed paired or unpaired *t*-test). The asterisks in/on each bar indicate significant differences from each ear of the wild-type **(B)** or control treatment **(E–G)**.

While isolated CD8^+^ T_RM_ cells in CHS-healed C57BL/6 mouse skin have been shown to secrete IFNγ upon an overnight re-stimulation (11), the identities of the cytokines secreted by CD8^+^ and CD4^+^ T_RM_ cells early after the re-challenge that initiated the LSM response remain unknown. We attempted to clarify this using comparisons of the early phase (0, 1.5, 3, and 6 h) mRNA expression of several cytokines in whole ear extracts after the re-challenge (Figure 8A and Supplemental Figure 7A). The levels of *Ifng, Tnf*, *Il4*, and *Il13* were higher than those in the naïve ears within 3 h of the re-challenge, and thus these cytokines were the candidate early responsible genes that initiate the LSM response. The levels of *Il1b, Il9, Il22*, and the neutrophil chemoattractants *Cxcl1* and *Cxcl2* were higher in the healed ears than in the naïve ears at more than 3 h after the re-challenge, suggesting that these are downstream effectors. The levels of *Il6, Il17a/f*, *Il18, Il33*, and *Tslp* were similar between the naïve and the healed ears (Supplemental Figure 7A).

We compared the expression of the above genes at 6 h after the re-challenge between WT BALB/c and DO11.10 mice lacking LSM. All genes in both ears of DO11.10 mice showed similar expression levels, with most being similar to or lower than the levels present in WT naive ears (Figure 8B). This result supports the notion that T cells accumulating in the healed ears of DO11.10 mice cannot respond to TNCB, and the expression of downstream effector genes and the LSM response are not induced without the secretion of the early responsible genes.

The naïve and the healed ears that were not treated (no stimulation) or re-challenged at 3.5 h before (1% TNCB) were subjected to enzymatic digestion with brefeldin A for 3.5 h for intracellular cytokine analyses (Figures 8C,D and Supplemental Figure 7B). Similar to T_RM_ cells forcibly activated with ionomycin + PMA during enzymatic digestion, a cell population strongly producing IFNγ and/or TNF emerged in both T_RM_ cells in the re-challenged healed ears, with more effective production being observed in CD8^+^ T_RM_ cells. The CD4^+^ T cells in naïve ears did not produce large amounts of IFNγ or TNF when re-challenged. In contrast, CD4^+^ and CD8^+^ T_RM_ cells in the healed ears did not produce IL-4 (Figure 8D). These results suggest that CD4^+^ and CD8^+^ T_RM_ cells in the healed ears both produced IFNγ and TNF early after the re-challenge.

To clarify whether these cytokines were required for the initiation of the LSM response, we simultaneously injected CHS-healed mice with anti-IFNγ and TNF-neutralizing mAbs with the re-challenge. However, the treatment had no or only a small inhibitory effect on the swelling response in both ears at 24 h after the re-challenge (Figure 8E). An injection with an irrelevant mAb (ACK2) showed that mAb reached all dermal tissues, but not some epidermal regions in the re-challenged healed ears (Supplemental Figure 7C), suggesting that mAb-mediated neutralization is ineffective for epidermal CD8^+^ T_RM_ cells. Therefore, we used Rux (a JAK1/JAK2 inhibitor, the downstream effectors of many cytokine receptors including IFNγ) and BAY (a NF-κB inhibitor, the downstream effectors of the TNF superfamily, including TNF and IL-1). The injection of BAY induced diarrhea at 10 mg/kg and death at 20 mg/kg on the next day, and 5 mg/kg BAY + 10 mg/kg Rux also induced diarrhea. Thus, we injected mice with 6 mg/kg BAY + 8 mg/kg Rux (a dose that is not harmful to mouse health) in addition to the topical application of 0.3% BAY + 0.5% Rux to the ears at 2 h before the re-challenge. This treatment weakly suppressed ear swelling in both the naïve and the healed ears on the next day; however, the LSM response persisted (Figure 8F). Therefore, we examined whether massive neutrophil accumulation in the healed ears in the early phase of the LSM response (at 4 h, the mice are alive) may be inhibited by injecting a lethal dose (20 mg/kg) of inhibitors (Figure 8G and Supplemental Figure 7D). Rux alone had no effect and BAY alone significantly inhibited the accumulation of neutrophils in the healed ears. The combined injection of Rux and BAY more effectively suppressed neutrophil accumulation in both ears. These results suggest that JAKs and NF-κB signaling cooperatively function in the LSM response.

### CD4^+^ T_RM_ Cells Are Maintained, While CD8^+^ T_RM_ Cells Disappear Over Time

**Figure 9:**
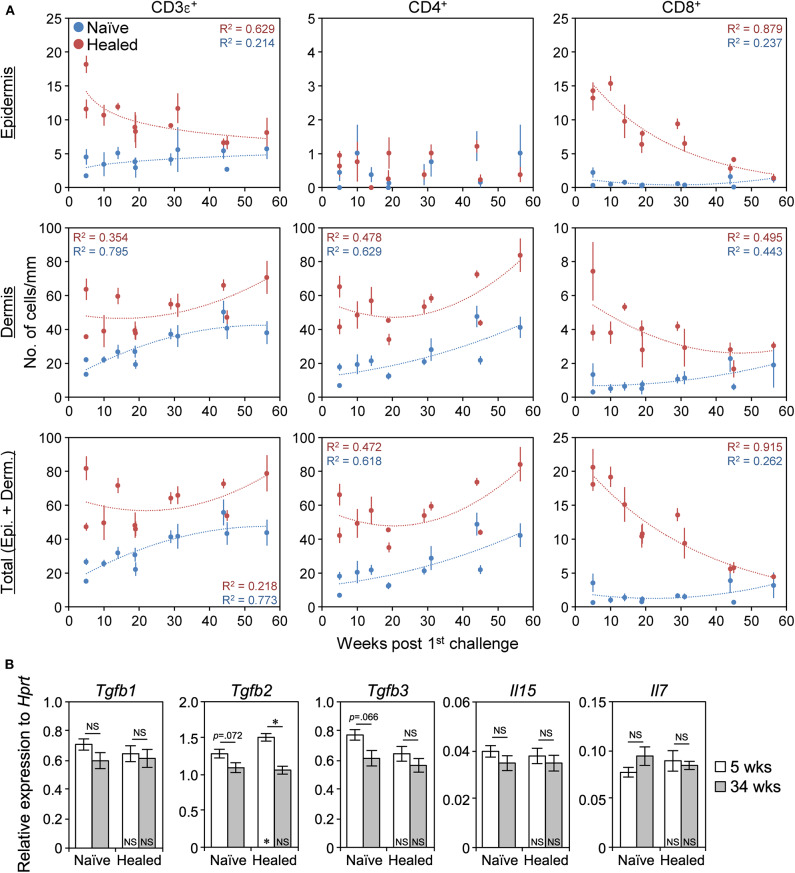
CD4^+^ T_RM_ cells are maintained, whereas CD8^+^ T_RM_ disappear over time. **(A)** The numbers of CD3ε^+^, CD4^+^, and CD8αβ^+^ cells along the cartilage in naïve and healed ear skin sections at 5–56 weeks (35–394 days) after the 1^st^ challenge with 1% 2,4,6,-trinitrochlorobenzene as assessed by IHC analyses were plotted. Approximate curves were selected according to the highest *R*^2^ values. 5 weeks/day 35 (*n* = 3 and *n* = 5), 10 weeks/day 70 (*n* = 3), 14 weeks/day 98 (*n* = 3), 19 weeks/day 133 (*n* = 6), 29 weeks/day 203 (*n* = 6), 31 weeks/day 217 (*n* = 3), 44 weeks/day 308 (*n* = 5), 45 weeks/day 315 (*n* = 5), and 56 weeks/day 394 (*n* = 3). Data represent means ± SE. Individual data are available in Supplemental Figure 8A. **(B)** The mRNA levels of the indicated genes in whole naïve and healed ear extracts of BALB/c mice at 5 weeks (day 37) and at 34 weeks (day 239) after the 1^st^ challenge as assessed by qRT-PCR (*n* = 4 each). Data represent means ± SE. *P < 0.05 (two-tailed paired or unpaired *t*-test). The asterisks in each bar indicate significant differences from each naïve ear.

Since LSM is a long-term memory (Figure 1C), we investigated whether T_RM_ cells were maintained in the healed ears over a prolonged period. The epidermal and the dermal CD8^+^ T_RM_ cells in the healed ears both gradually disappeared over time and reached the same levels as those in the naïve ears between 45 and 56 weeks (Figure 9 and Supplemental Figures 8A,B), as reported in C57BL/6 mice (11). In contrast, the healed ears contained more CD4^+^ T_RM_ cells than the naïve ears at every time point by 56 weeks after the 1^st^ challenge, and their numbers in both ears persisted and even increased throughout that period (Figure 9 and Supplemental Figures 8A,B). The CD4^+^ T_RM_ cells always exclusively localized in the dermis and outnumbered the CD8^+^ T_RM_ cells. The ratio of Treg cells in the naïve and the healed ears did not markedly change even after 30 weeks (Supplemental Figure 3C). The IF analysis of the naïve and the healed ear skin sections on day 394 (56 weeks) showed that, similar to that of day 35 (Figure 2D), the CD8^+^ cells were exclusively CD3^+^, while 90% of CD4^+^ cells were CD3^+^ (Supplemental Figure 8C). These results indicated that the LSM response in BALB/c mice was initially generated by both CD4^+^ and CD8^+^ T_RM_ cells after the resolution of the 1^st^ CHS (at 5 weeks) and was then mediated by CD4^+^ T_RM_ cells after more than 45 weeks because of the gradual loss of CD8^+^ T_RM_ cells over time.

We then investigated the expression of genes required for the survival of T_RM_ cells to explain the gradual loss of CD8^+^ T_RM_ cells. Transforming growth factor β (TGFβ) and IL-15 signaling are involved in CD8^+^ T_RM_ survival, and IL-7 signaling is involved in CD4^+^ T_RM_ survival (20, 21, 42). The expression of IL-2/15 receptor β (IL-2/15Rβ) and IL-7Rα on CD4^+^ and CD8^+^ T cells in the naïve and the healed skin remained unchanged between 5 and 30 weeks after the 1^st^ challenge (Supplemental Figure 8D). The mRNA levels of *Tgfb1/3, Il15*, and *Il7* in the whole ear extract were also unchanged between the naïve and the healed ears and between 5 and 34 weeks after the 1^st^ challenge (Figure 9B). The expression of *Tgfb2* in the healed ears was slightly lower at 34 weeks than at 5 weeks but was still prominent (Figure 9B). These results suggest that the gradual loss of CD8^+^ T_RM_ cells may not be solely explained by the loss of these molecules from T_RM_ cells or skin.

## Discussion

ACD/CHS-healed skin in humans and animals forms LSM, which increases local antigen sensitivity and causes disease relapse. ACD-healed human skin dominantly has persisting CD4^+^ T cells in the dermis (35); however, CHS-healed C57BL/6 mouse skin predominantly has increased numbers of CD8^+^ T_RM_ cells in the epidermis that are responsible for the formation of LSM (10, 11). It currently remains unclear whether CD4^+^ T_RM_ cells contribute to the formation of LSM and flare-up reactions upon antigen re-exposure. We herein demonstrated that the CHS-healed skin of BALB/c mice contained increased numbers of CD4^+^ and CD8^+^ T_RM_ cells, with a predominance of CD4^+^ T_RM_ cells. Moreover, we showed not only that CD4^+^ or CD8^+^ T_RM_ cells redundantly mediated the LSM response (the flare-up) upon the re-challenge but also that long-term LSM was subsequently mediated by CD4^+^ T_RM_ cells over time because of the gradual loss of CD8^+^ T_RM_ cells. Thus, the present results provide evidence to support the targeting of CD4^+^ T_RM_ cells, in addition to CD8^+^ T_RM_ cells, in order to prevent flare-up reactions of human ACD.

In BALB/c CHS-healed skin, increased numbers of CD4^+^ T cells almost exclusively resided in the dermis, as shown in ACD-healed human skin (35), while CD8^+^ T cells largely resided in the epidermis, as shown in CHS-healed C57BL/6 mouse skin (11), suggesting that BALB/c mice have a combination of the traits of the C57BL/6 mouse and human skin in the context of LSM. Our result showing that the skin CD4^+^ or CD8^+^ T_RM_ cells mediate the flare-up of CHS upon antigen re-exposure indicates that the flare-up is initiated within the epidermis by MHC class I-regulated LSM or within the dermis by MHC class II-regulated LSM. Thus, there is mechanistic redundancy and robustness in the formation of LSM. Moreover, our results suggest that both systems use the same molecular cascade to induce the flare-up reaction, namely, the rapid production of IFNγ and TNF from each T_RM_ cell, which induced downstream molecules (such as IL-1, IL-9, and Cxcl1/2) produced by many other cell types in the skin (2, 43–45).

Similar to that in C57BL/6 mice (11), the CD8^+^ T_RM_ cells in the CHS-healed skin of BALB/c mice gradually decreased in number over time. This decline has also been shown in skin CD8^+^ T_RM_ cells generated from antigen-specific TCR-transgenic T cells by a viral infection or unspecific stimulation (34, 46, 47); however, the decrease observed in these cases was very sharp (many cells were lost within several weeks) and a small population stabilized and persisted. The non-recirculating property of skin CD8^+^CD69^+^CD103^+^ T_RM_ cells in previously inflamed skin has repeatedly been demonstrated (25, 30, 33, 34, 48), suggesting that loss occurs within the skin. The present results showed that the mRNA expression of the T_RM_ survival factor TGFβ2 slightly decreased in the healed ear skin over time; however, the mechanisms regulating the gradual loss of CD8^+^ T_RM_ cells from the healed skin have not yet been elucidated in detail.

In contrast to their CD8^+^ counterparts, increased CD4^+^ T_RM_ cell numbers in BALB/c CHS-healed skin persisted over time. However, it currently remains unclear whether they are a truly resident, non-migrating population. In the naïve skin, the vast majority of CD4^+^ T cells are recirculating and in equilibrium with the circulation despite their surface expression of CD69 and CD103 (29). In previously inflamed skin, heterogeneity has been reported in the increased number of CD4^+^ T cells, with both recirculating and resident populations being detected (29, 31), and the sustained increase in CD4^+^ T cell numbers was attributed to the enhanced recruitment of memory cells from the circulation as well as the formation of the long-persisting T_RM_ population (29). Nevertheless, the non-recirculating property of the increased number of skin CD4^+^ T cells with the T_RM_ phenotype (CD69^+^) in the short term (~2 weeks) generated after skin pathogen infections has been demonstrated (25, 31). Moreover, residence is a dominant feature of CD4^+^CD69^+^ memory T cells generated by viral infection in various non-lymphoid tissues (49). Since the majority of CD4^+^ T cells in the BALB/c CHS-healed skin were CD69^+^ cells that were resistant to antibody-mediated cell depletion, their sustained increase may result from their long lifespan and persistence within the skin. However, we cannot rule out the possibility that, during the long-term resting state after the resolution of CHS, some CD4^+^ T_RM_ cells in the BALB/c healed skin down-regulate the expression of T_RM_ markers, exit the skin, reenter the circulation, and eventually migrate to the distal skin and reassume the T_RM_ phenotype, as reported previously for human skin (24).

The swelling response of both the naïve and the healed ears upon a re-challenge increased over time after the resolution of the 1^st^ CHS (Figure 1C). The increases in CD4^+^ T_RM_ cell numbers in the ear skin over time (Figure 9) may be related to heightened responsiveness. However, if this is the case, the mechanisms by which TNCB-specific skin CD4^+^ T_RM_ cells increase in number during the resting state, particularly in the naïve skin, and how this leads to stronger swelling intensities in both ears upon the re-challenge at 56 weeks than in the healed ears at 5 weeks after the 1^st^ challenge currently remain unclear. Further studies that consider the possibility of T_RM_ cell relocation (24) and the enhanced recruitment of memory cells (29) are needed to obtain a more detailed understanding of this phenomenon.

The mechanisms responsible for the differential formation of CD4^+^ and CD8^+^ T_RM_ cells in the CHS-healed skin of C57BL/6 and BALB/c mice have yet to be clarified. Moreover, the formation of CD4^+^ T_RM_ cells in the C57BL/6 healed skin differs with CHS protocols and appears to require repeated hapten applications or intense inflammation for effective formation, with the CD8^+^ T_RM_ cells dominating in many cases (9–11). Therefore, the C57BL/6 mice appear to produce more skin CD8^+^ T_RM_ cells than CD4^+^ T_RM_ cells in CHS, while the BALB/c mice are the opposite. However, the inflammation-healed skin of C57BL/6 mice was found to produce similar or higher numbers of CD4^+^ T_RM_ cells than CD8^+^ T_RM_ cells following a skin infection with *Candida albicans* (31) or the cowpox virus (25). These findings suggest that C57BL/6 mice retain the ability to form large numbers of skin CD4^+^ T_RM_ cells under different settings. Thus, different regulatory mechanisms function in C57BL/6 and BALB/c mice to establish the balance between skin CD4^+^ and CD8^+^ T_RM_ cell formation in the context of CHS.

In addition to ACD, growing evidence points to the involvement of T_RM_ cells in inflammatory skin disorders frequently relapsing in the same locations, such as atopic dermatitis, fixed drug eruption, psoriasis, and vitiligo (13, 15, 16, 50, 51). Pathogenic CD4^+^ and/or CD8^+^ T cells with the T_RM_ phenotype have been shown to remain in the remitting skin after treatments for these disorders (23, 52–54). In some settings, remitting skin after years of treatment still contains elevated numbers of CD4^+^ and CD8^+^ T cells (52, 55), suggesting that the human skin CD4^+^ T_RM_ cells also have a long lifespan and that CD8^+^ T_RM_ cells do not always decrease in number after resolution. Based on the present results, the CD8^+^ and CD4^+^ T_RM_ cells in the remitting skin may form LSM for each disease, create an antigen-sensitive state, and induce local flare-up reactions upon re-exposure to antigens.

The present results highlight the important contribution of skin CD4^+^ T_RM_ cells to the formation of LSM and the initiation of flare-up reactions in ACD/CHS. Thus, the removal of pathogenic CD4^+^ T_RM_ cells, in addition to CD8^+^ T_RM_ cells, has the potential to break the cycle of relapsing–remitting skin diseases.

## Data Availability Statement

The datasets generated for this study are available on request to the corresponding author.

## Ethics Statement

The animal study was reviewed and approved by Animal Care and Use Committee of Tottori University.

## Author Contributions

AM designed and performed the research. AM and S-IH analyzed the data and wrote the manuscript.

## Conflict of Interest

The authors declare that the research was conducted in the absence of any commercial or financial relationships that could be construed as a potential conflict of interest.
