# Supplementary figures and images for: CD4+ Resident Memory T Cells Mediate Long-Term Local Skin Immune Memory of Contact Hypersensitivity in BALB/c Mice
Source: Front Immunol. 2020 May 19;11:775. doi: 10.3389/fimmu.2020.00775 (PMC7248184; doi:10.3389/fimmu.2020.00775)

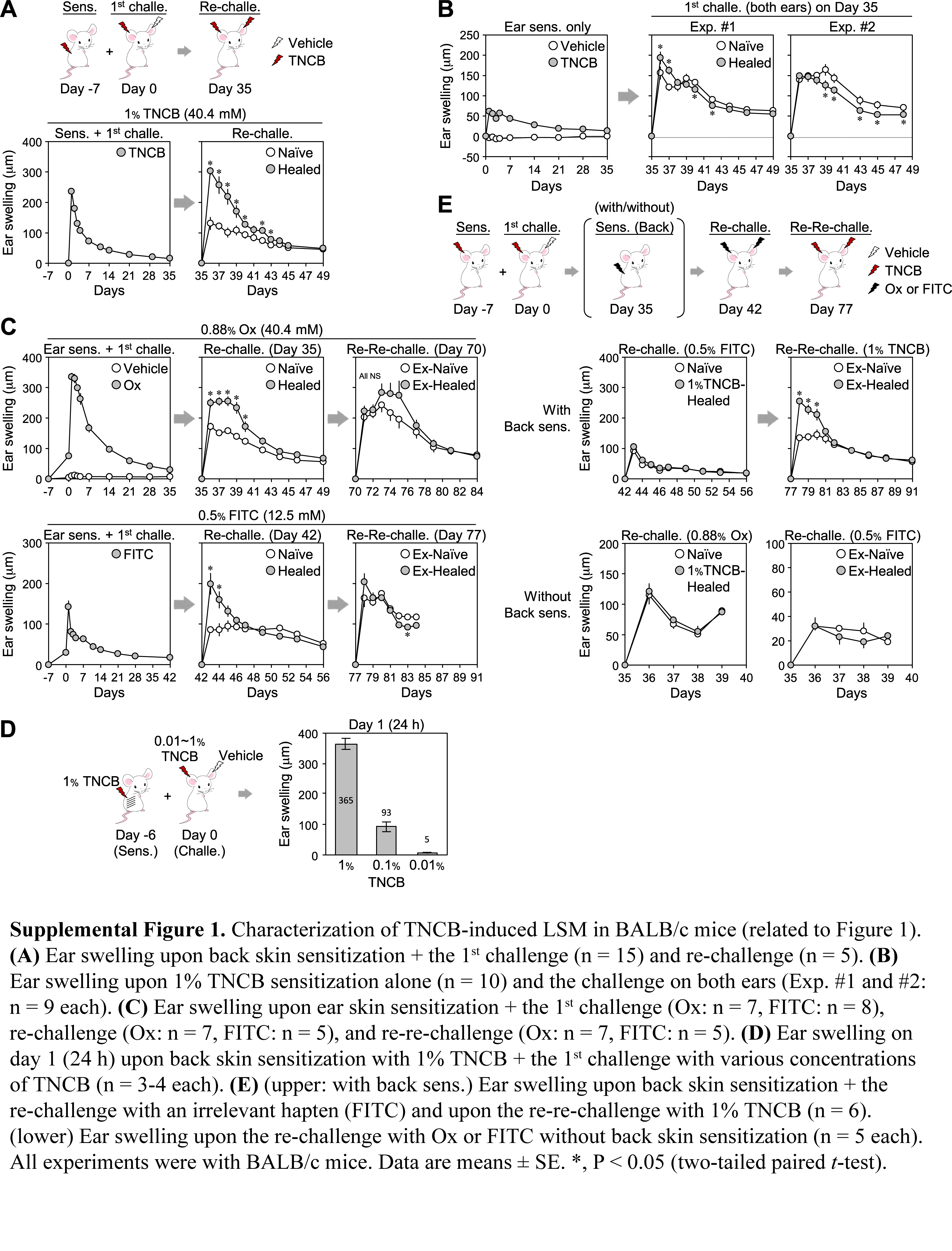

Supplement: Supplementary file 1 [file Image_1.TIF]

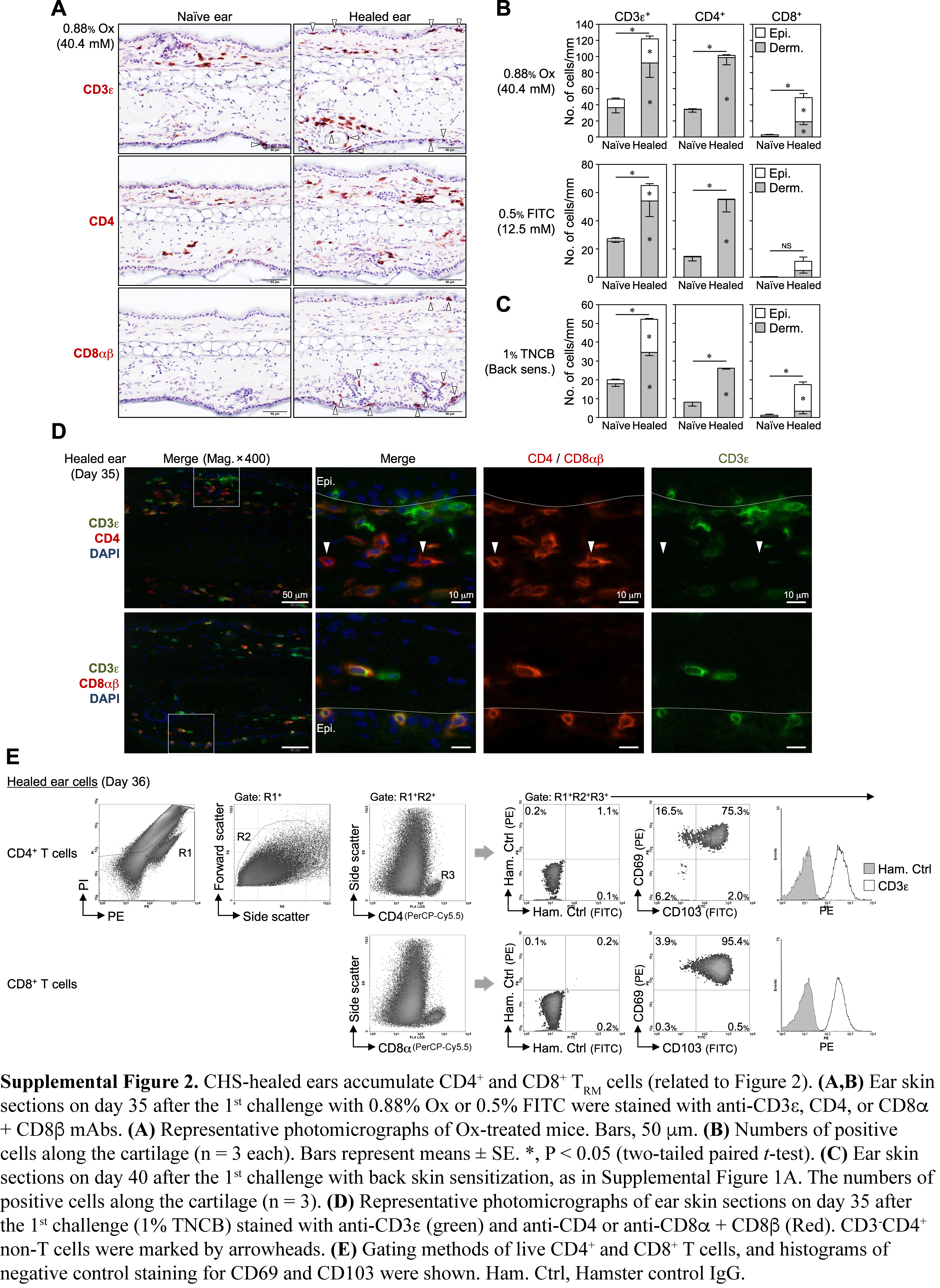

Supplement: Supplementary file 2 [file Image_2.TIF]

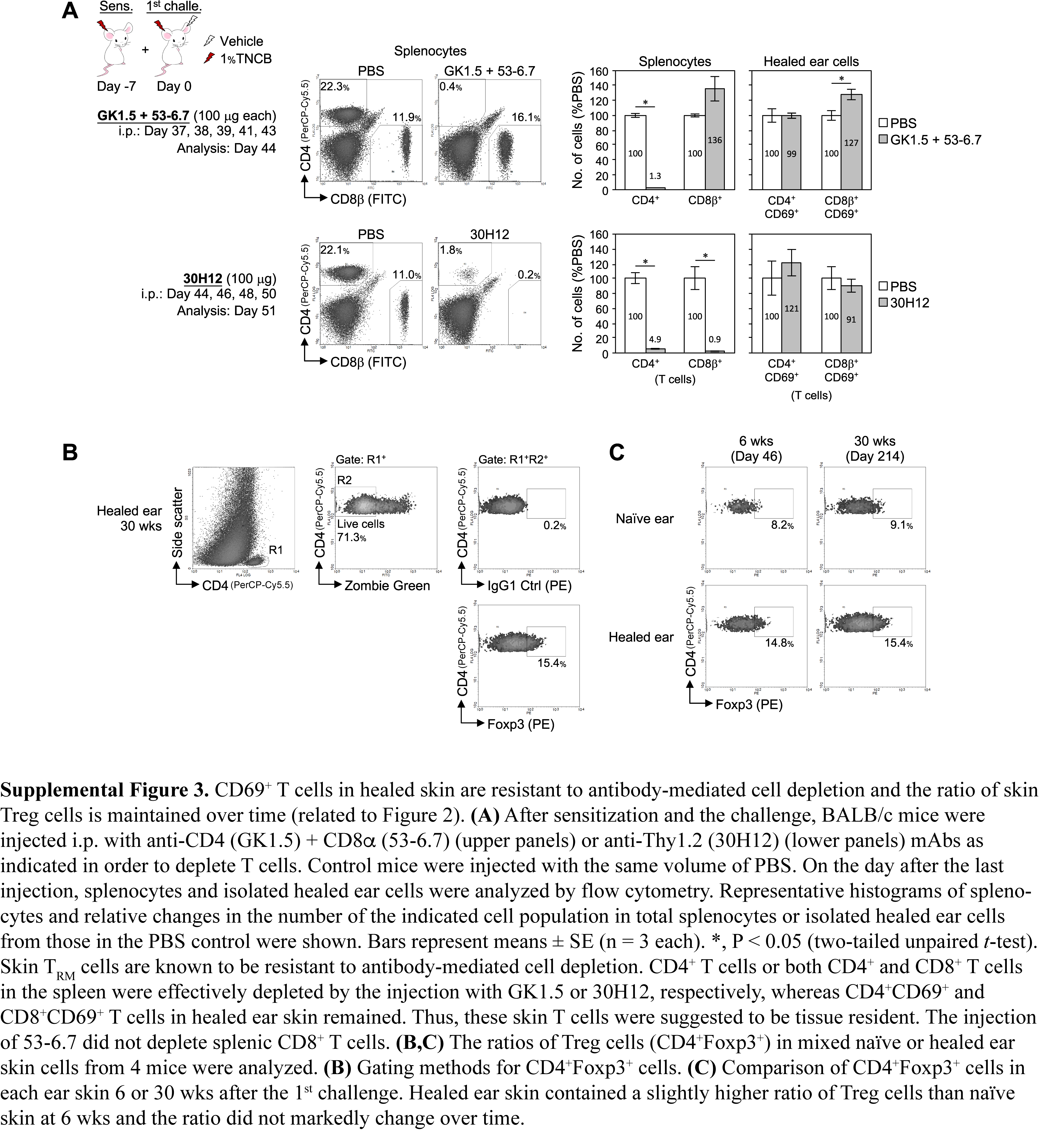

Supplement: Supplementary file 3 [file Image_3.TIF]

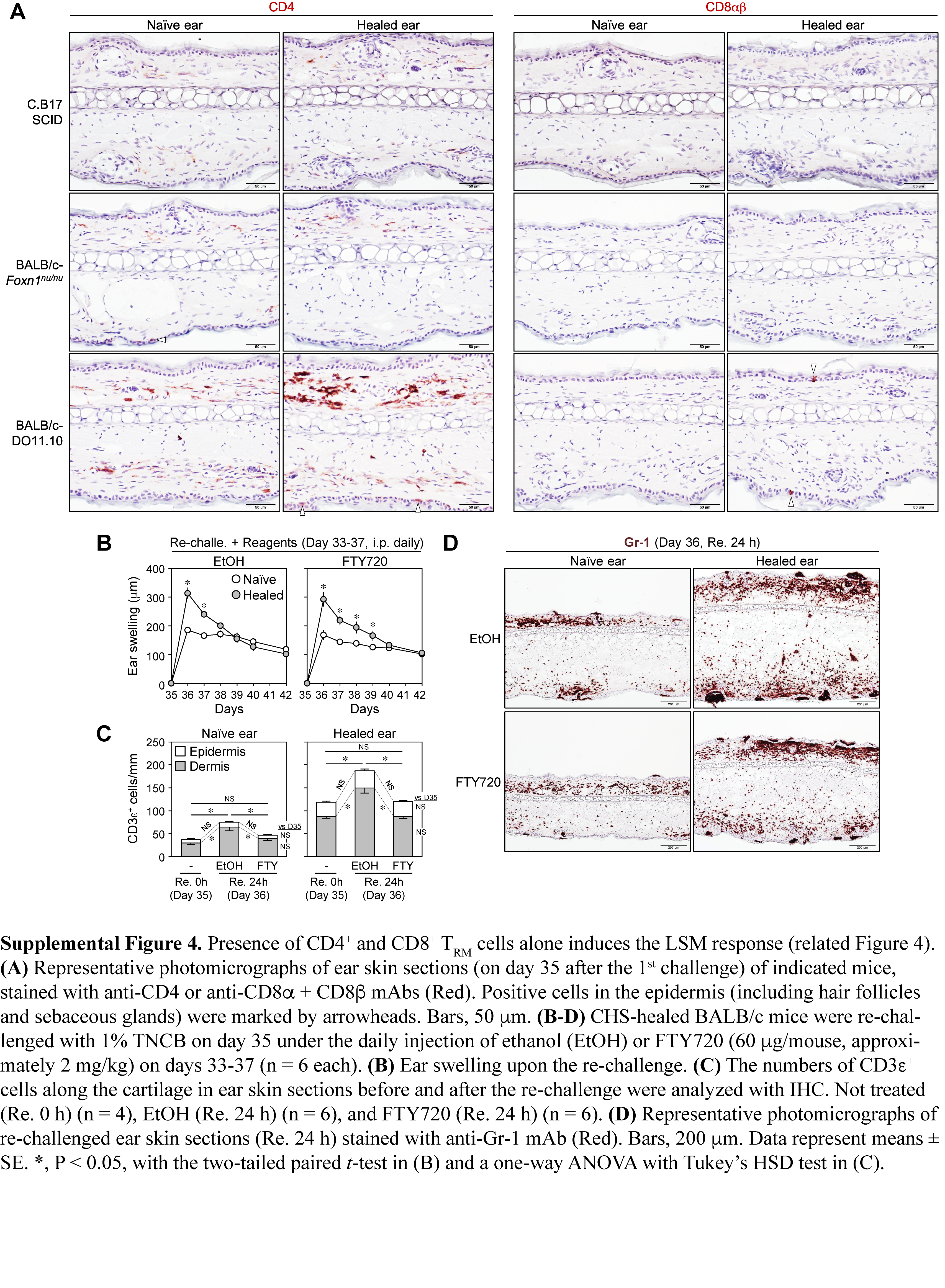

Supplement: Supplementary file 4 [file Image_4.TIF]

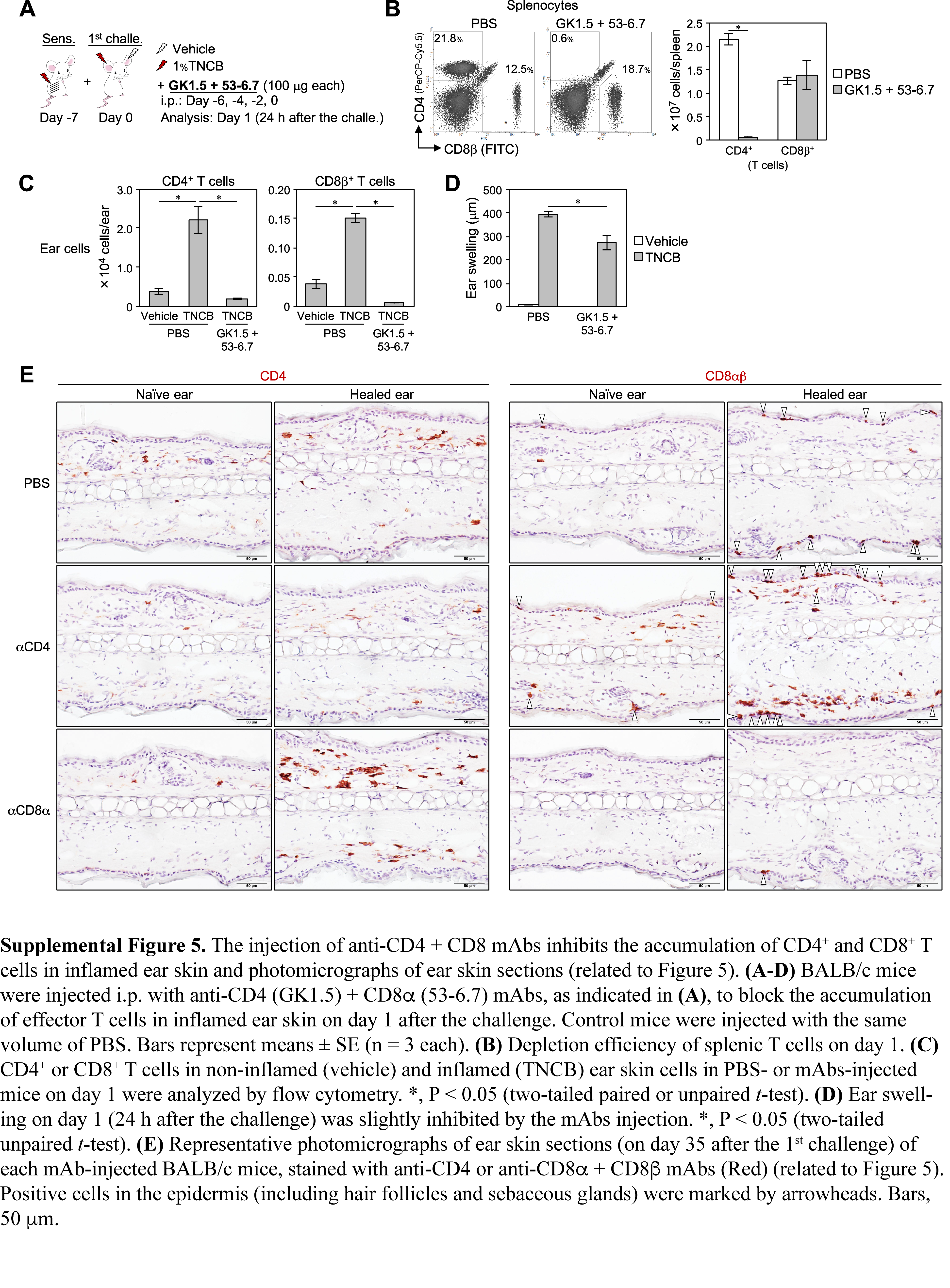

Supplement: Supplementary file 5 [file Image_5.TIF]

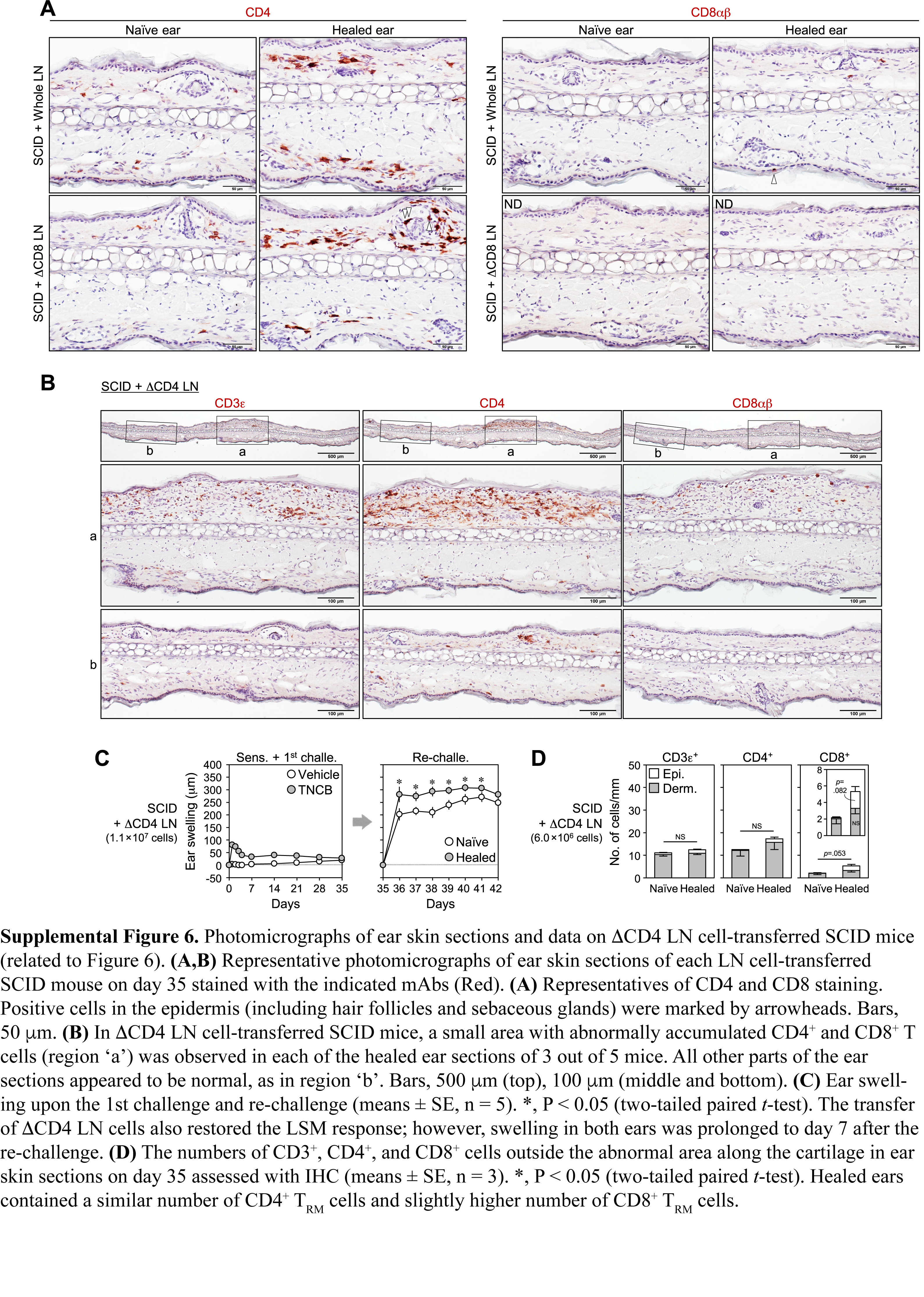

Supplement: Supplementary file 6 [file Image_6.TIF]

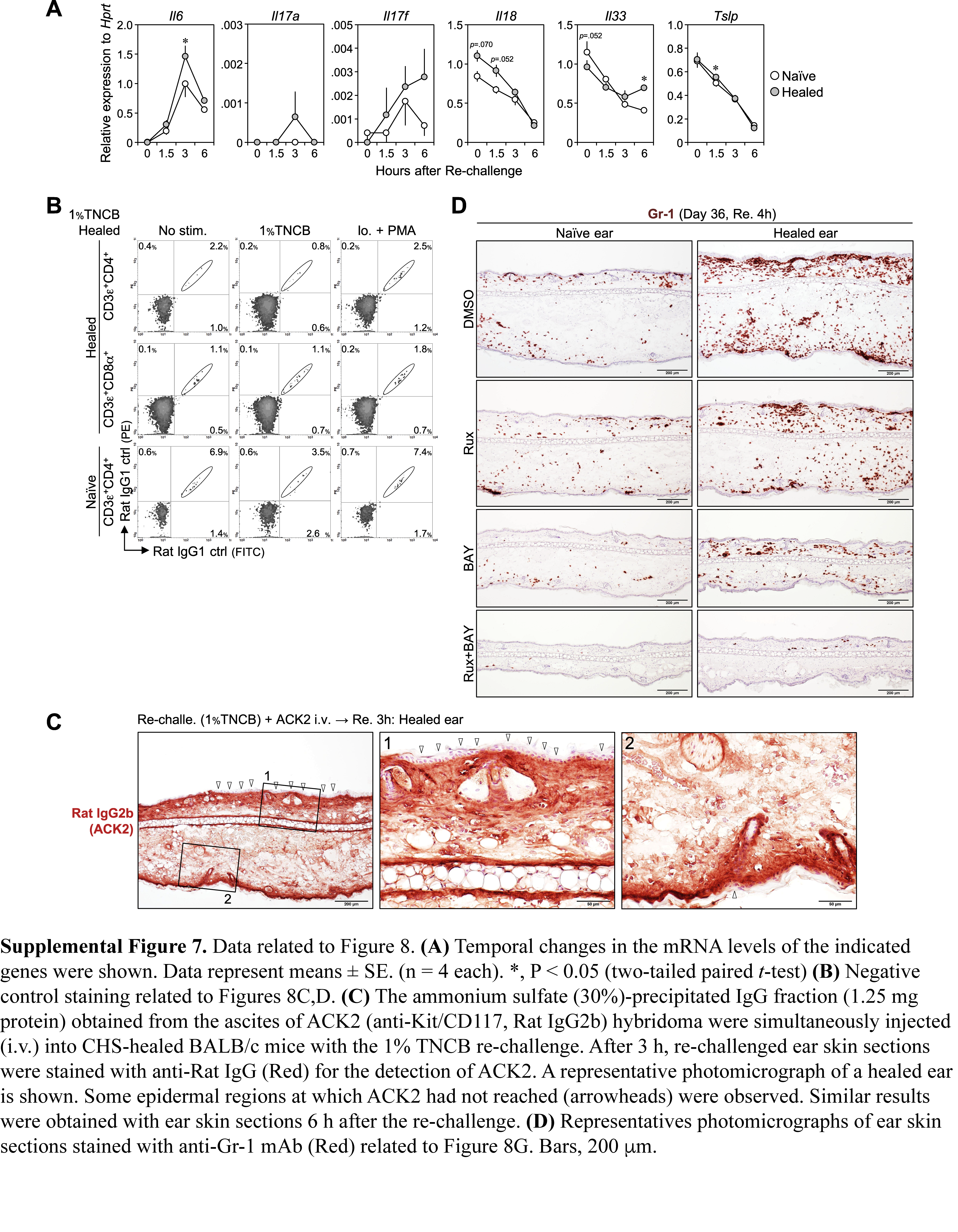

Supplement: Supplementary file 7 [file Image_7.TIF]

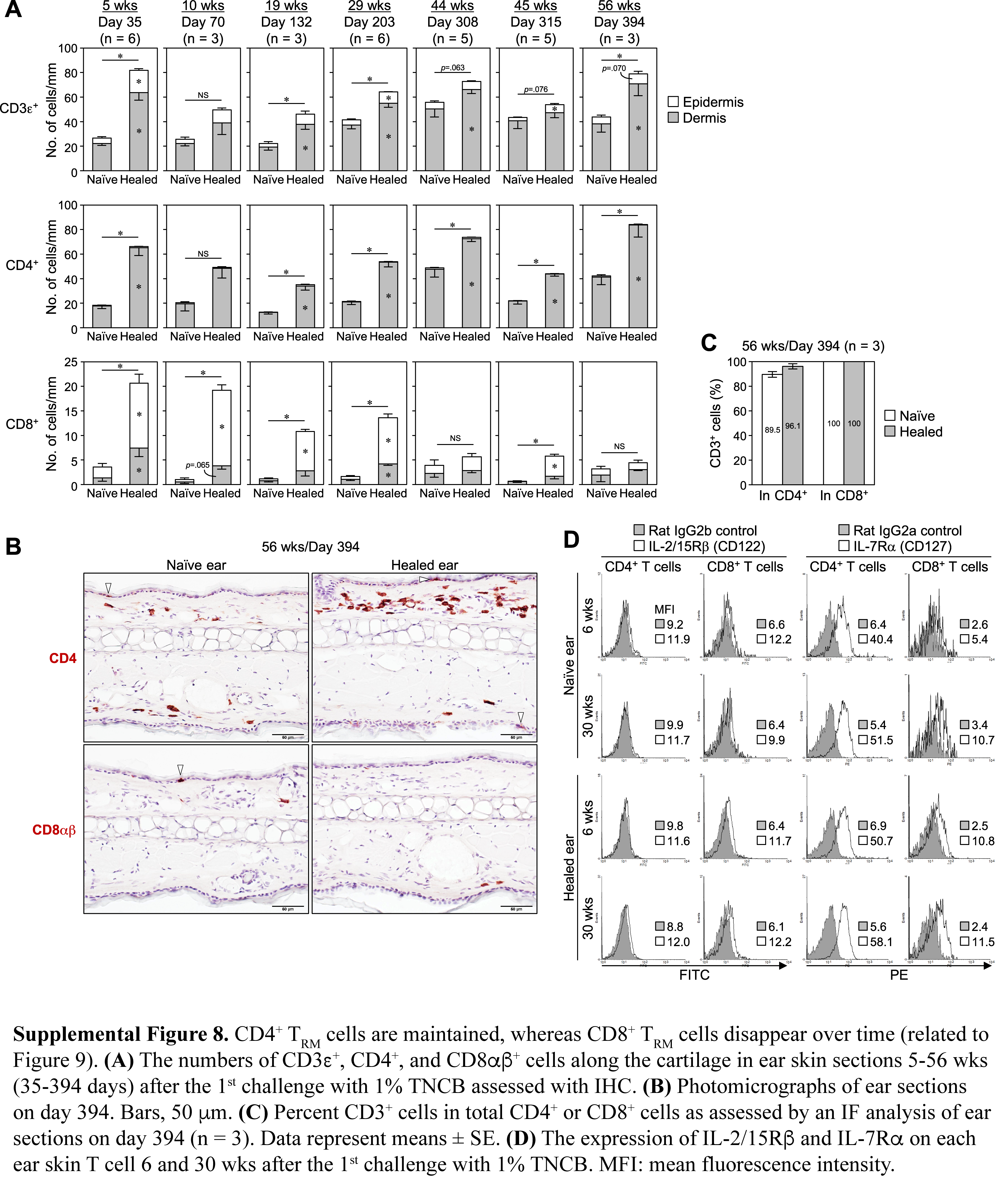

Supplement: Supplementary file 8 [file Image_8.TIF]
